# The mRNA and protein of IL-8 oppositely regulate PRRSV replication via nucleoprotein and 14-3-3γ

**DOI:** 10.1128/jvi.00655-25

**Published:** 2025-07-24

**Authors:** Yingchao Li, Jiaqi Liu, Dexin Li, Mingyu Dou, Chaolun Fu, Ting Chen, Hongyan Gao, Man Lu, Yang Shen, Pingping Yang, Yanmeng Hou, Hongjie Yuan, Yumei Cai, Baoquan Li, Yihong Xiao

**Affiliations:** 1 Department of Fundamental Veterinary Medicine, College of Veterinary Medicine, Shandong Agricultural University 34734, Tai'an, Shandong, China; Loyola University Chicago - Health Sciences Campus, Maywood, Illinois, USA

**Keywords:** PRRSV, 14-3-3γ, IL-8 mRNA, IL-8 protein, N protein, viral replication

## Abstract

Porcine reproductive and respiratory syndrome virus (PRRSV) has posed a significant threat to the global swine industry for over 3 decades, resulting in substantial economic losses. In this study, we identified the cellular protein 14-3-3γ as an antiviral factor that suppresses PRRSV replication by downregulating the mRNA levels of interleukin-8 (IL-8). Intriguingly, while IL-8 mRNA levels were positively correlated with PRRSV genome replication, the IL-8 protein itself exhibited a potent inhibitory effect on viral replication. Mechanistic studies revealed that 14-3-3γ enhances IL-8 transcription. In contrast, the viral nucleocapsid (N) protein upregulates IL-8 transcription. A critical nuclear localization signal (NLS) domain (amino acid residues 11–13) within the N protein, particularly the lysine residues at positions 10 and 12, was identified as essential for this regulatory function. The nuclear distribution of the N protein was found to be indispensable for its activity. Furthermore, we demonstrated that 14-3-3γ and IL-8 competitively bind to the N protein, modulating its nuclear distribution. This study elucidates the intricate regulation of IL-8 at both transcriptional and protein levels in modulating PRRSV replication. It uncovers a novel function of IL-8 in viral pathogenesis and highlights its potential as a promising candidate for the development of anti-PRRSV therapeutics.

**IMPORTANCE:**

Porcine reproductive and respiratory syndrome virus (PRRSV) has severely impacted swine production worldwide for decades. This study reveals that the cellular protein 14-3-3γ suppresses PRRSV replication by downregulating interleukin-8 mRNA, while IL-8 protein itself inhibits viral replication. Mechanistically, 14-3-3γ upregulates IL-8 transcription, whereas the viral nucleocapsid (N) protein enhances IL-8 transcription, which is dependent on its nuclear localization signal (amino acid residues 11–13, particularly K10/K12). Nuclear distribution of the N protein is essential for this function. Additionally, 14-3-3γ and IL-8 competitively bind N protein, modulating its nuclear trafficking. This dual regulation of IL-8 at transcriptional and protein levels highlights its antiviral role and potential as a therapeutic target against PRRSV.

## INTRODUCTION

Porcine reproductive and respiratory syndrome virus (PRRSV) is a major causative agent of porcine reproductive and respiratory syndrome (PRRS), a disease that inflicts significant economic losses on the global swine industry. Annual economic losses attributed to PRRS are estimated at 6.25–15.25 USD per pig (1). PRRSV belongs to the family *Arteriviridae* and the order *Nidovirales* and is classified into two genotypes: PRRSV-1 (European type) and PRRSV-2 (North American type) (2). The disease is characterized by mild to severe respiratory symptoms in infected piglets and growing pigs, as well as reproductive failure in pregnant sows. PRRSV exhibits considerable genetic and virulence variability among isolates. Since its emergence, several highly pathogenic PRRSV (HP-PRRSV) strains have evolved, leading to acute disease outbreaks in various countries, particularly in China. These include the evolution of low-pathogenicity strains into highly pathogenic variants, such as NADC30-like, NADC34-like, and numerous recombinant strains (3–5). In the USA, highly virulent strains, including 1-8-4 and 1-4-4, have been reported (6, 7). The lack of effective vaccines and therapeutics continues to make PRRS a significant challenge for the swine industry.

PRRSV is an enveloped, single-stranded, positive-sense RNA virus. Its genome encodes two large nonstructural polyproteins (pp1a and pp1ab), four membrane-associated glycoproteins (GP2a, GP3, GP4, and GP5), three unglycosylated membrane proteins (E, open reading frame 5a, and M), and a nucleocapsid (N) protein (8–10). The N protein, encoded by ORF7, is a multifunctional viral protein essential for genome encapsulation and virion assembly. It is also the most abundant viral protein in infected cells (11). The N protein plays critical roles in viral replication, viral entry, and modulation of the host immune response (12–14). It is a serine-phosphoprotein that localizes to both the cytoplasm and nucleus due to the presence of nuclear localization signals (NLS) at amino acid positions 10–13 and 41–47 (15–17). These NLS regions are closely associated with PRRSV replication (18); however, the precise role of the N protein in viral replication remains poorly understood.

PRRSV negatively regulates the host immune response, leading to persistent immunosuppression. The PRRSV N protein plays a key role in this process by inhibiting the induction of type I interferons (IFNs), including IFN-β, and suppressing the phosphorylation and nuclear translocation of interferon regulatory factor 3 (19). Tripartite motif protein 25 (TRIM25), a known inhibitor of PRRSV replication, is antagonized by the N protein through competitive binding. The N protein disrupts TRIM25-mediated retinoic acid-inducible gene-I ubiquitination, thereby suppressing IFN-β production (20). These findings underscore the role of the N protein in counteracting host antiviral responses. However, it remains unclear whether the nuclear localization of the N protein is linked to its ability to modulate IFN signaling.

Cytokines play a central role in the host immune response to viral infections, serving as critical mediators of both antimicrobial activity and inflammation. PRRSV infection in swine triggers a severe inflammatory response, upregulating the expression of proinflammatory cytokines such as interleukin-1β (IL-1β), IL-12, IL-8, and tumor necrosis factor-α (TNF-α) both *in vitro* and *in vivo* (21–24). The rapid production of these cytokines is closely associated with the hallmark pathological changes of severe interstitial pneumonia observed in PRRSV-infected animals. Notably, IL-8 is upregulated following PRRSV infection and has been shown to facilitate viral replication (25). Studies have demonstrated that serum levels of key cytokines, including IL-8, IL-1β, and IFN-γ, are strongly correlated with PRRSV infection, accounting for approximately 84% of the observed variations (23, 26). IL-8, produced by various cell types such as monocytes and endothelial cells, is a potent mediator of proinflammatory activities (27–29). Studies highlight its proinflammatory role, showing that monoclonal antibodies targeting IL-8 significantly reduce clinical disease activity in conditions such as palmoplantar pustulosis (30). While the involvement of the N protein in the production of cytokines such as IL-8, IL-10, and IL-15 has been reported (12, 13, 31), the specific roles of these cytokines in PRRSV pathogenesis remain poorly understood.

In this study, we uncovered a dual role for IL-8 in PRRSV infection, revealing distinct effects of IL-8 mRNA and IL-8 protein on viral replication. We further elucidated the underlying mechanisms driving this phenomenon. Our findings suggest that the IL-8 protein may serve as a promising therapeutic target for combating PRRSV infection.

## RESULTS

### 14-3-3γ is an antiviral factor during PRRSV replication

**Fig 1:**
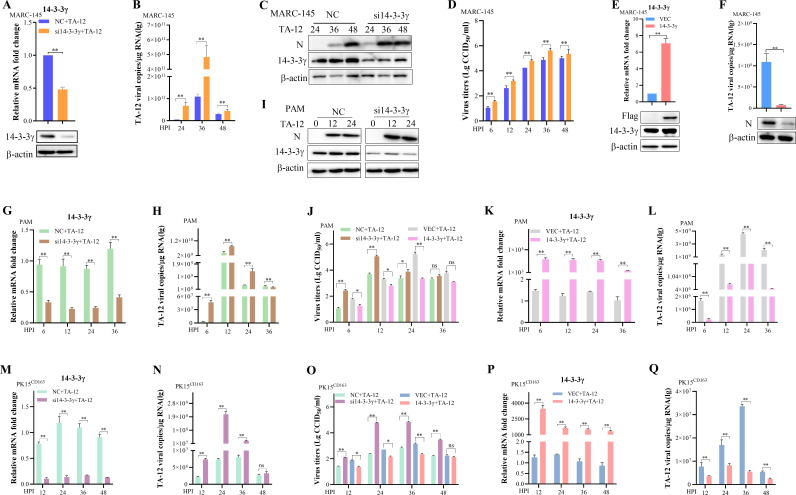
14-3-3γ functions as an anti-PRRSV factor. 14-3-3γ knockdown promotes PRRSV replication. MARC-145 cells (A–D), PAMs (G–J), and PK15^CD163^ cells (M–O) were transfected with siRNA targeting 14-3-3γ or control siRNA (NC) and then infected with TA-12. Cell samples were collected at the indicated HPI. The mRNA levels of 14-3-3γ and N were quantified by qPCR, and protein levels were detected by Western blotting. Viral titers in the supernatant were measured. Overexpression of 14-3-3γ inhibits PRRSV replication. MARC-145 cells (**E and F**), PAMs (**K and L**), and PK15^CD163^ cells (P and Q) were transfected with 3×Flag-14-3-3γ or an empty vector and then infected with TA-12. Cell samples were collected at the indicated HPI. Total RNA and protein were extracted and analyzed by qPCR and Western blotting, respectively. Viral titers in the supernatant were measured. Glyceraldehyde-3-phosphate dehydrogenase (GAPDH) was used as an internal control for qPCR, and β-actin was used as an internal control for Western blotting. Error bars represent the SD of three experimental replicates. Asterisks indicate significant differences compared to controls: *P < 0.05; *P* < 0.01 (unpaired two-tailed Student’s *t*-test).

Previous studies have suggested that 14-3-3γ plays a role in PRRSV replication (32, 33); however, its specific function remained unclear. To investigate the role of 14-3-3γ in PRRSV infection, 14-3-3γ was knocked down using specific small interfering RNA (siRNA) in the PRRSV-permissive MARC-145 cell line (Fig. 1A), followed by infection with the HP-PRRSV strain TA-12 for 24, 36 and 48 hours. Quantitative real-time PCR (qPCR) and Western blot analyses revealed that knockdown of 14-3-3γ enhanced TA-12 infection (Fig. 1B and C). Consistent with these findings, viral titers in the supernatant of 14-3-3γ-knockdown MARC-145 cells were significantly increased (Fig. 1D). To further validate these results, 14-3-3γ was overexpressed in MARC-145 cells. Both qPCR and Western blot analyses demonstrated that overexpression of 14-3-3γ suppressed TA-12 infection (Fig. 1E and F). To extend these observations to primary cells, 14-3-3γ was knocked down in porcine alveolar macrophages (PAMs) (Fig. 1G). Similar to the results in MARC-145 cells, knockdown of 14-3-3γ in PAMs led to increased viral genome replication, protein expression (Fig. 1H and I), and viral titers (Fig. 1J). Conversely, overexpression of 14-3-3γ in PAMs significantly reduced TA-12 genome replication (Fig. 1K and L) and viral titers (Fig. 1J). To further corroborate these findings, the role of 14-3-3γ in PRRSV infection was examined using the PK15^CD163^ cell line. Knockdown of 14-3-3γ in PK15^CD163^ cells promoted TA-12 infection (Fig. 1M and N) and increased viral titers (Fig. 1O). In contrast, overexpression of 14-3-3γ in PK15^CD163^ cells inhibited TA-12 genome replication (Fig. 1P and Q) and reduced viral titers (Fig. 1O). Collectively, these results demonstrate that 14-3-3γ acts as an antiviral factor during PRRSV infection.

### 14-3-3γ downregulates the mRNA level of IL-8 during PRRSV replication

**Fig 2:**
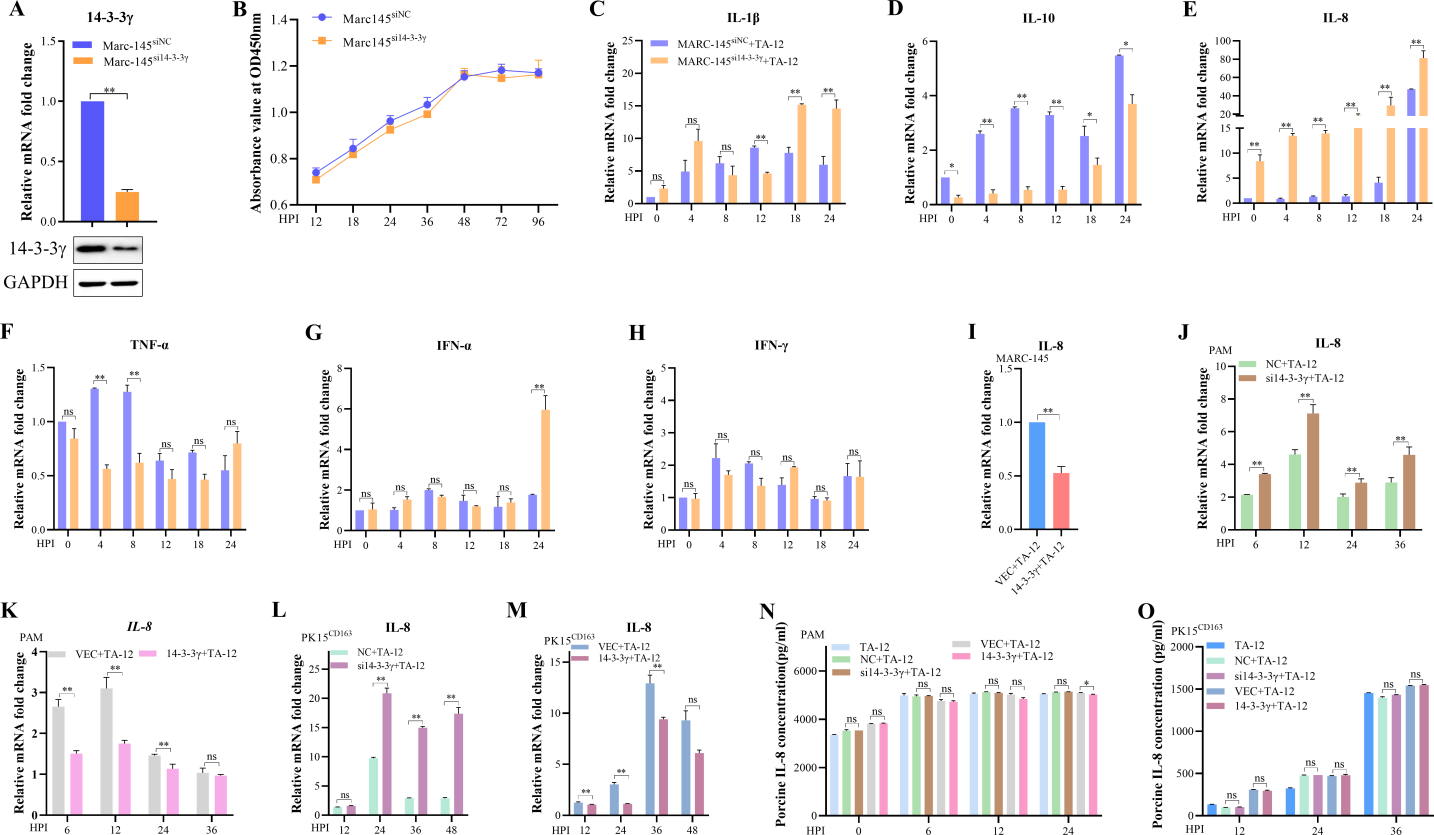
14-3-3γ downregulates PRRSV infection-induced IL-8 mRNA levels. (**A**) Identification of the 14-3-3γ-knockdown cell line, MARC-145^si14-3-3γ^, by qPCR and Western blotting. (**B**) Cell growth kinetics of MARC-145^siNC^ and MARC-145^si14-3-3γ^. Cells were plated in 24-well plates, and at 12, 18, 24, 36, 48, 72, and 96 h post-plating, the Cell Counting Kit-8 reagent was added. After a 3 h incubation at 37°C, cell counting was performed by measuring absorbance at 450 nm. (C–H) Effect of 14-3-3γ on the expression of PRRSV-induced cytokines. TA-12 was inoculated into MARC-145^siNC^ and MARC-145^si14-3-3γ^ cells. Cell samples were collected at 0, 4, 8, 12, 18, and 24 HPI, and total RNA was extracted. The mRNA levels of IL-1β (**C**), IL-10 (**D**), IL-8 (**E**), TNF-α (**F**), IFN-α (**G**), and IFN-γ (**H**) were measured by relative qPCR. (**I**) MARC-145 cells were transfected with 3×Flag-14-3-3γ or an empty vector and then infected with TA-12. Cell samples were collected at 24 HPI, and IL-8 mRNA levels were measured by relative qPCR. (**J, L**) 14-3-3γ knockdown upregulates IL-8 mRNA. PAMs (**J**) and PK15^CD163^ cells (**L**) were transfected with siRNA targeting 14-3-3γ or non-targeting control (NC) for 24 h and then infected with HP-PRRSV TA-12 at an MOI of 0.01. IL-8 mRNA levels were detected by qPCR. (**K, M**) Overexpression of 14-3-3γ downregulates IL-8 mRNA. PAMs (**K**) and PK15^CD163^ cells (**M**) were transfected with 3×Flag-14-3-3γ or an empty vector and then infected with TA-12. IL-8 mRNA levels were detected by qPCR. (**N, O**) 14-3-3γ protein has no effect on IL-8 protein levels. 14-3-3γ was knocked down or overexpressed in PAMs (**N**) and PK15^CD163^ cells (**O**), followed by infection with TA-12. Cell supernatants were collected at the indicated HPI, and IL-8 protein levels were measured by ELISA.Glyceraldehyde-3-phosphate dehydrogenase (GAPDH) was used as an internal control for qPCR, and β-actin was used as an internal control for Western blotting. Multiplicity of infection (MOI), enzyme-linked immunosorbent assay (ELISA). Error bars represent the SD of three experimental replicates. Asterisks indicate significant differences compared to controls: *P < 0.05; *P* < 0.01 (unpaired two-tailed Student’s *t*-test).

Accumulated evidence has shown that 14-3-3γ proteins are involved in the inflammatory response (34, 35). PRRSV dysregulates cytokine levels to subvert the host immune response and facilitate its infection (21–25). These findings prompted us to investigate the role of 14-3-3γ in cytokine expression during PRRSV infection. To obtain comparable data, a 14-3-3γ-knockdown MARC-145 (MARC-145^si14-3-3γ^) cell line was established, along with a scramble negative control (MARC-145^siNC^) (Fig. 2A). Knockdown of 14-3-3γ had no effect on cell growth (Fig. 2B). The mRNA level of cytokines, including IL-1β, IL-10, IL-8, TNF-α, IFN-α, and IFN-γ, was detected in MARC-145^si14-3-3γ^ and MARC-145^siNC^ cells following infection with TA-12. Knockdown of 14-3-3γ upregulated IL-1β at 18 and 24 HPI (Fig. 2C). The mRNA levels of IL-10 were downregulated at 0, 4, 8, 12, and 18 HPI, while TNF-α mRNA levels were downregulated at 4 and 8 HPI (Fig. 2C and D). Notably, knockdown of 14-3-3γ significantly upregulated IL-8 mRNA levels at all time points in MARC-145^si14-3-3γ^ (0, 4, 8, 12, 18, and 24 HPI) (Fig. 2E). In contrast, no significant changes were observed in the mRNA levels of IFN-α or IFN-γ (Fig. 2F through H). These results suggest that 14-3-3γ plays a key role in regulating IL-8 during PRRSV replication, prompting further investigation.

To further validate the role of 14-3-3γ in regulating IL-8 mRNA levels, 14-3-3γ was overexpressed in MARC-145 cells. Overexpression of 14-3-3γ downregulated the IL-8 mRNA levels (Fig. 2I). Similarly, knockdown or overexpression of 14-3-3γ in PAMs revealed that IL-8 mRNA levels were upregulated in 14-3-3γ-knockdown PAMs (Fig. 2J) and downregulated in 14-3-3γ-overexpressing PAMs (Fig. 2K). To further corroborate these findings, 14-3-3γ was knocked down in the PK15^CD163^ cells, and the cells were infected with TA-12. qPCR results showed that IL-8 mRNA levels were upregulated in the 14-3-3γ-knockdown cells but not in control cells (transfected with NC) (Fig. 2L) and were downregulated in 14-3-3γ-overexpressing cells (Fig. 2M).

The IL-8 protein level in the supernatant of PAMs was measured by enzyme-linked immunosorbent assay (ELISA). Interestingly, no changes in IL-8 protein levels were observed in the supernatant of 14-3-3γ-knockdown or 14-3-3γ-overexpressing PAMs compared to the control (PAMs transfected with NC) (Fig. 2N). To further confirm the effect of 14-3-3γ on IL-8 protein levels, the supernatants of PK15^CD163^ cells were also analyzed by ELISA. Similarly, no changes in IL-8 protein levels were detected in the supernatant of 14-3-3γ-knockdown or 14-3-3γ-overexpressing PK15^CD163^ cells (Fig. 2O). These results indicate that knockdown of 14-3-3γ upregulates the mRNA level of IL-8 but does not affect its protein level.

### 14-3-3γ downregulates the mRNA level of IL-8 to inhibit PRRSV replication

**Fig 3:**
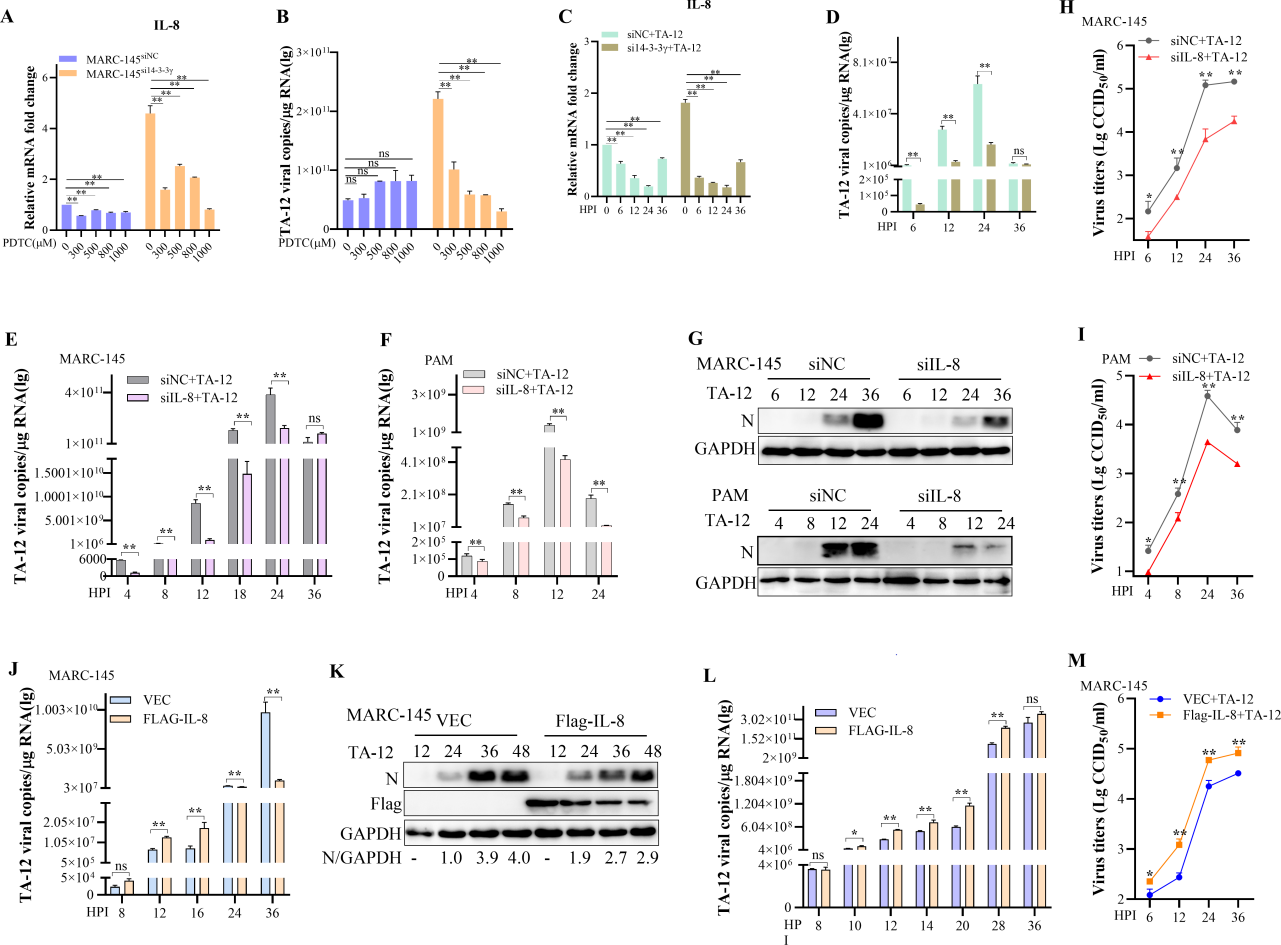
IL-8 mRNA positively correlates with PRRSV replication. (A–D) PDTC decreases both IL-8 mRNA levels and viral genome replication. MARC-145^siNC^ and MARC-145^si14-3-3γ^ cells were pretreated with 0, 300, 500, 800, and 1,000 µM PDTC for 1 h and then inoculated with TA-12. Cell samples were collected at 24 HPI, and the mRNA levels of IL-8 (**A**) and the viral genome (**B**) were detected by qPCR. PAM cells were transfected with siRNA targeting 14-3-3γ or control siRNA (NC) and then pretreated with 300 µM PDTC for 1 h. Cell samples were collected after infection with TA-12 at 0, 6, 12, 24, and 36 HPI. The mRNA levels of IL-8 (**C**) and the viral genome (**D**) were detected by qPCR. (E–I) Knockdown of IL-8 inhibits PRRSV replication. MARC-145 cells (**E, G, H**) and PAMs (**F, G, I**) were transfected with siRNA targeting IL-8 or control siRNA (NC) and then infected with TA-12. Cell samples were collected at the indicated HPI. TA-12 infection was evaluated based on the N gene by qPCR, Western blotting, and viral titration. (J–M) PRRSV replication is closely related to IL-8 mRNA and protein levels. MARC-145 cells were inoculated with TA-12 for 2 h and then transfected with 3×Flag-IL-8 or an empty vector. Cell samples were collected at the indicated HPI. The viral genome was detected by qPCR (**J**) and Western blotting (**K**). MARC-145 cells were inoculated with PRRSV, and 3×Flag-IL-8 was transfected at 4, 6, 8, 10, 16, 24, and 32 HPI. After 4 h of each transfection, cells and supernatants were collected. The PRRSV genome was detected by qPCR (**L**), and viral titers in the supernatants were measured (**M**). Glyceraldehyde-3-phosphate dehydrogenase (GAPDH) was used as an internal control for qPCR. Error bars represent the SD of three experimental replicates. GAPDH was used as an internal control for Western blotting. Asterisks indicate significant differences compared to controls: *P < 0.05; *P* < 0.01 (unpaired two-tailed Student’s *t*-test).

The above results indicates that 14-3-3γ acts as an antiviral factor during PRRSV replication and downregulates the PRRSV-induced mRNA level of IL-8. To confirm the relationship between IL-8 mRNA levels and TA-12 infection, pyrrolidinedithiocarbamate ammonium (PDTC), an inhibitor of nuclear factor kappa-B (NF-κB) that attenuates IL-8 production and suppresses the accumulation of IL-8 mRNA (36), was added to MARC-145^si14-3-3γ^ and MARC-145^siNC^ cell lines. qPCR results showed that PDTC decreased IL-8 mRNA levels in both MARC-145^si14-3-3γ^ and MARC-145^siNC^ cells (Fig. 3A). Correspondingly, viral genome levels were reduced only in MARC-145^si14-3-3γ^ cells (Fig. 3B).

PDTC was also added to PAMs with or without 14-3-3γ knockdown prior to TA-12 inoculation. The results demonstrated that PDTC downregulated IL-8 expression, significantly blocking TA-12 infection (Fig. 3C and D). However, no significant changes were observed in the control group. These findings suggest that IL-8 mRNA levels are positively correlated with viral genome levels and that 14-3-3γ inhibits TA-12 replication by downregulating IL-8 mRNA.

To further validate these results, IL-8 was knocked down in MARC-145 cells and PAMs. Western blot, qPCR, and viral titration results showed that reduced IL-8 mRNA levels were negatively correlated with PRRSV replication (Fig. 3E through I).

To further investigate the role of IL-8 mRNA in PRRSV infection, TA-12-infected MARC-145 cells were transfected with a construct containing the IL-8 gene. qPCR results revealed that viral infection increased before 16 HPI but decreased after 24 HPI, coinciding with the appearance of IL-8 protein (Fig. 3J and K). To specifically examine the effect of IL-8 mRNA on PRRSV replication, the IL-8 gene construct was transfected into TA-12-infected MARC-145 cells for 4 h, a time point at which IL-8 mRNA was overexpressed but no IL-8 protein was detected. qPCR and viral titration results demonstrated that IL-8 mRNA significantly increased PRRSV infection (Fig. 3L and M). These results indicate that IL-8 mRNA levels are positively correlated with viral genome levels, suggesting that IL-8 mRNA may facilitate PRRSV replication.

### IL-8 mRNA positively correlates with PRRSV genome independent of its open reading frame

**Fig 4:**
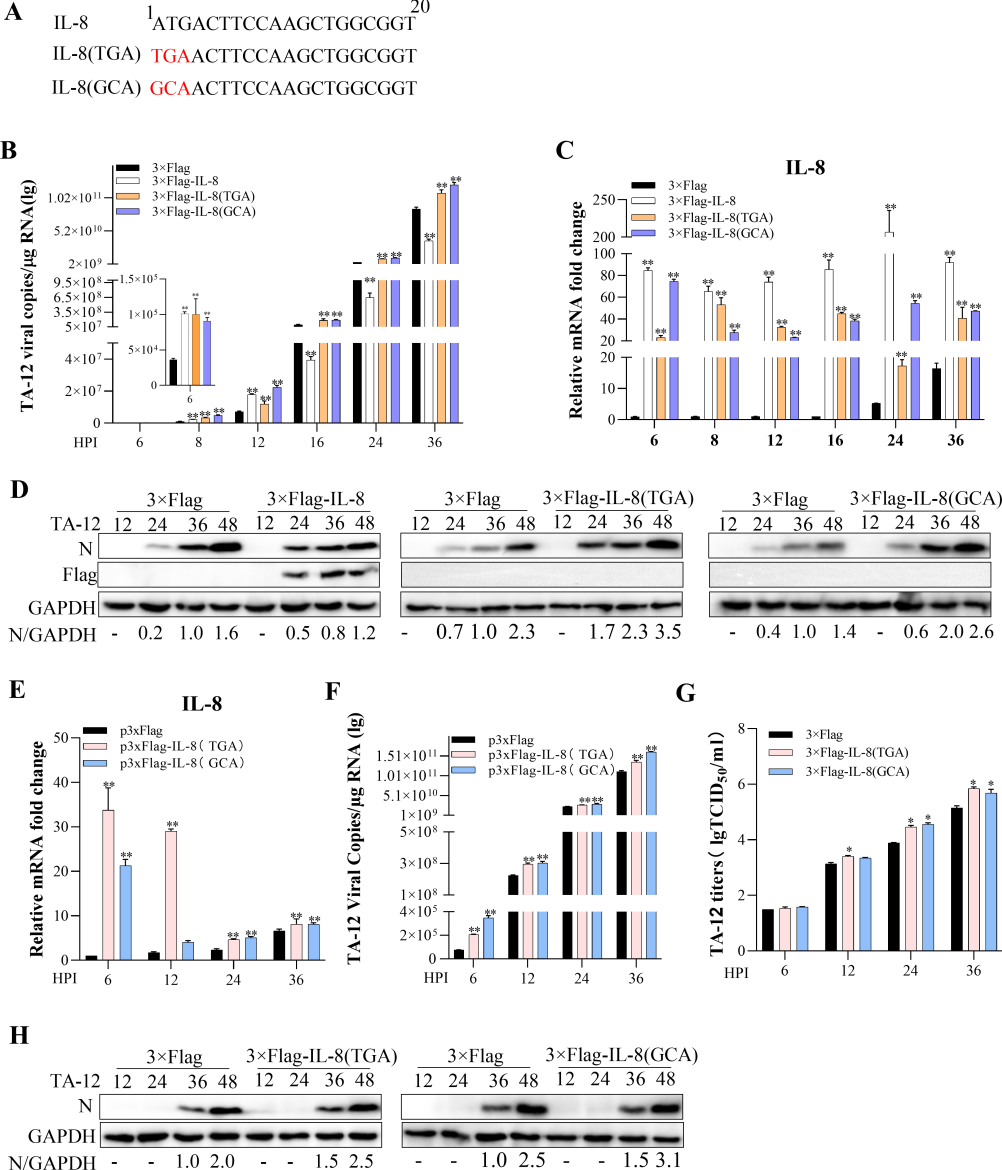
IL-8 mRNA positively correlates with PRRSV replication independent of its open reading frame. (**A**) Sequence of mutant IL-8 mRNA. (B–D) Mutant IL-8 mRNA positively correlates with PRRSV replication (method 1). MARC-145 cells were infected with TA-12 for 2 h and then transfected with 3×Flag-IL-8, 3×Flag-IL-8 (TGA), 3×Flag-IL-8 (GCA), or an empty vector. Cell samples were collected at the indicated HPI. The TA-12 genome was detected by qPCR (**B**), the level of IL-8 mRNA was measured by qPCR (**C**), and viral protein levels were assessed by Western blotting (**D**). (E–H) Mutant IL-8 mRNA positively correlates with PRRSV replication (method 2). MARC-145 cells were transfected with 3×Flag-IL-8, 3×Flag-IL-8 (TGA), 3×Flag-IL-8 (GCA), or an empty vector for 12 h and then inoculated with TA-12. Cells were collected at the indicated HPI. The level of IL-8 mRNA was measured by qPCR (**E**), the TA-12 genome was detected by qPCR (**F**), viral titers were determined (**G**), and viral protein levels were assessed by Western blotting (**H**). Glyceraldehyde-3-phosphate dehydrogenase (GAPDH) was used as an internal control for qPCR. Error bars represent the SD of three experimental replicates. GAPDH was used as an internal control for Western blotting. Asterisks indicate significant differences compared to controls: *P < 0.05; *P* < 0.01 (unpaired two-tailed Student’s *t*-test).

The above results demonstrates that IL-8 mRNA levels are positively correlated with PRRSV genome levels. To further investigate the role of the ORF in IL-8 mRNA, the translation initiation codon (ATG) was mutated to TGA or GCA (Fig. 4A). The TGA mutation introduces a premature stop codon, abolishing the translation of IL-8 protein while preserving the RNA sequence. The GCA mutation (a synonymous substitution at the third codon position) was designed to maintain the RNA structure but prevent initiation of translation. These mutations allowed us to dissect the functional contribution of IL-8 mRNA itself, independent of its protein product. Two experimental approaches were employed to examine the role of mutant IL-8 mRNA in PRRSV replication. First, MARC-145 cells were infected with TA-12, and the IL-8 mutants were transfected at 2 HPI. The results showed that transfection of the IL-8 mutants led to high levels of mutant IL-8 mRNA, as well as increased PRRSV genome levels and protein expression (Fig. 4B through D). Second, MARC-145 cells were transfected with IL-8 mutants for 12 h to maintain high levels of IL-8 RNA, followed by inoculation with TA-12. The levels of mutant IL-8 RNA were positively correlated with PRRSV genome levels, viral titers, and protein expression (Fig. 4E through H). These results further confirm that IL-8 mRNA positively correlates with PRRSV replication, independent of its ability to be translated into protein. IL-8 protein blocks PRRSV replication.

### IL-8 protein inhibits PRRSV replication

**Fig 5:**
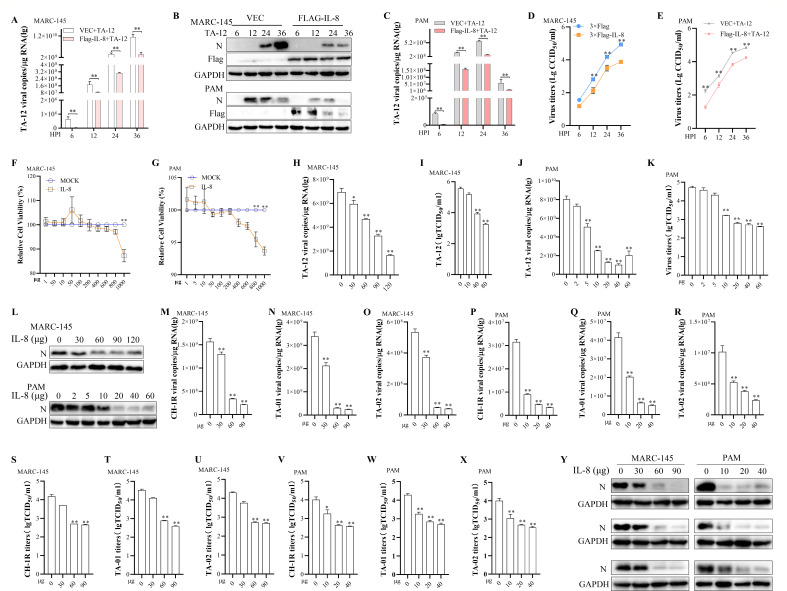
IL-8 protein inhibits PRRSV infection. (A–E) IL-8 transfection inhibits PRRSV replication. MARC-145 cells and PAMs were transfected with 3×Flag-IL-8 or an empty vector for 24 h and then infected with TA-12. Cell samples were collected at 6, 12, 24, and 36 HPI. The TA-12 genome was detected by qPCR (**A, C**), viral protein levels were measured by Western blotting (**B**), and viral titers were determined (**D, E**). (**F, G**) Cytotoxicity of IL-8 protein. The cytotoxicity of IL-8 protein was assessed using the Cell Counting Kit-8 (CCK-8) assay. Monolayers of MARC-145 cells (**F**) and PAMs (**G**) were treated with IL-8 protein at different concentrations for 24 h, followed by the addition of the CCK-8 reagent. Cell viability was measured by absorbance at 450 nm. (H–L) IL-8 protein inhibits TA-12 replication. MARC-145 cells and PAMs were inoculated with TA-12 for 2 h, and IL-8 protein at different concentrations was added. Cell samples were collected at 24 HPI. The TA-12 genome was detected by qPCR (**H, J**), viral titers were determined (**I, K**), and viral protein levels were measured by Western blotting (**L**). (M–Y) IL-8 protein inhibits replication of different PRRSV strains. MARC-145 cells and PAMs were infected with CH-1R, TA-01, and TA-02 (alongside TA-12) for 2 h, and IL-8 protein at different concentrations was added. Cells and supernatants were collected at 24 HPI. Viral loads were quantified by absolute qPCR targeting the **N** gene (M–R), and viral titers in the supernatants were determined by CCID_50_ assay (S–X). Viral protein levels were assessed by Western blotting (**Y**). Glyceraldehyde-3-phosphate dehydrogenase (GAPDH) was used as an internal control for qPCR. Error bars represent the SD of three experimental replicates. GAPDH was used as an internal control for Western blotting. Asterisks indicate significant differences compared to controls: *P < 0.05; *P* < 0.01 (unpaired two-tailed Student’s *t*-test).

The above results demonstrates that transfection of IL-8 is positively correlated with viral genome levels before 16 HPI but negatively correlated after 24 HPI (Fig. 3J and K). The results in Fig. 4D indicated that PRRSV genome and protein levels in cells transfected with mutant IL-8 mRNA (which cannot be translated) increased persistently throughout the course of infection. In contrast, in cells transfected with wild-type IL-8 mRNA, PRRSV replication decreased from 16 HPI. These findings suggest that IL-8 protein may play a distinct role in PRRSV replication. To investigate the role of IL-8 protein in PRRSV replication, a construct containing the IL-8 gene was transfected into MARC-145 cells and PAMs for 24 h to allow IL-8 protein expression, followed by inoculation with TA-12. The results showed that IL-8 protein levels were significantly negatively correlated with PRRSV genome levels, protein expression, and viral titers, indicating that IL-8 protein may inhibit PRRSV replication (Fig. 5A through E). To further confirm the role of IL-8 protein, IL-8 was expressed *in vitro*, purified, and tested for cytotoxicity (Fig. 5F and G). The renatured IL-8 protein was then added to MARC-145 cells and PAMs. The results demonstrated that IL-8 protein significantly reduced PRRSV genome levels, protein expression, and viral titers (Fig. 5H through L). The effect of IL-8 protein on different PRRSV strains was also evaluated. The results showed that IL-8 protein significantly inhibited the replication of CH-1R, TA-01, and TA-02 (Fig. 5M through Y). Collectively, these findings indicate that IL-8 protein can block PRRSV replication.

### N protein upregulates IL-8 transcription dependent on its nuclear localization

**Fig 6:**
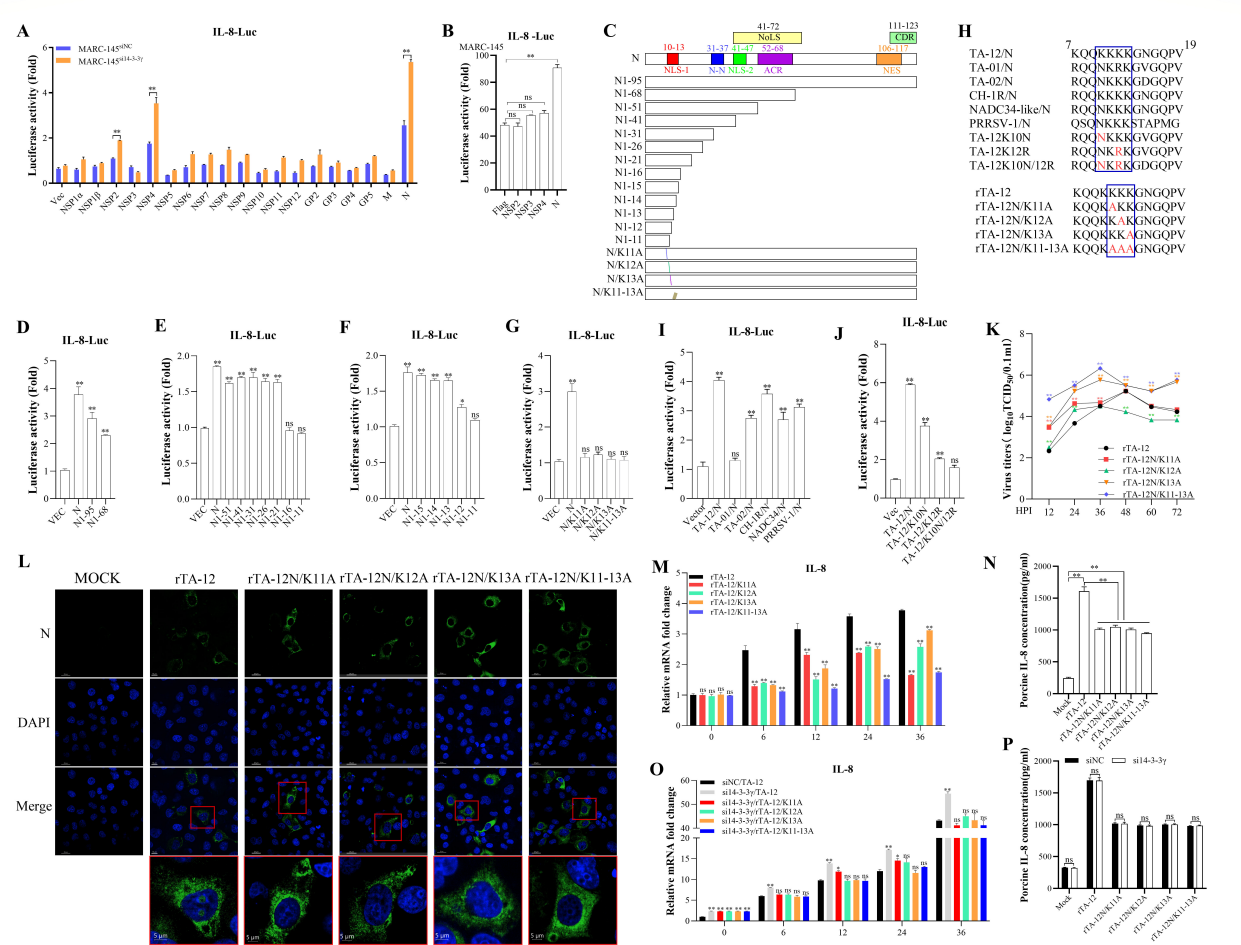
N protein upregulates IL-8 transcription dependent on its nuclear localization. (**A**) Viral proteins regulate IL-8 transcription. Constructs encoding viral proteins and pGL3-IL-8-Luc (containing the IL-8 promoter) were co-transfected into HEK293T cells for 24 h. Cells were collected, and firefly and Renilla luciferase activities were measured using a dual-luciferase reporter assay. (**B**) N protein activates IL-8 transcription. After co-transfection of NSP2, NSP3, NSP4, and N with pGL3-IL-8-Luc into MARC-145 cells, cells were collected, and firefly and Renilla luciferase activities were measured using a dual-luciferase reporter assay. (**C**) Schematic structure of truncated and mutant N proteins. (D–F) Identification of key sites in N regulating IL-8 transcription. Truncated mutants of N were co-transfected with pGL3-IL-8-Luc into 293T cells, and cells were collected for dual-luciferase reporter assays. (**G**) Role of N proteins from different PRRSV strains in regulating IL-8 transcription. N proteins from various PRRSV strains were co-transfected with pGL3-IL-8-Luc into 293T cells, and cells were collected for dual-luciferase reporter assays after 24 h. (**H**) Schematic diagrams of mutant N genes and PRRSV strains. (**I, J**) Identification of key residues in N regulating IL-8 transcription across PRRSV strains. Constructs containing mutant N genes were co-transfected with pGL3-IL-8-Luc into 293T cells, and cells were collected for dual-luciferase reporter assays after 24 h. (**K**) The multi-step growth curves of rescued mutant PRRSV strains. The rescued mutant viruses of passage 2 were inoculated into MARC-145 cells at an MOI of 0.01, respectively. Virus supernatants harvested at 12, 24, 36, 48, 60, and 72 HPI were titrated by median tissue culture infectious dose 50 (TCID_50_) assay. Each data point represents the mean ± deviation of duplicates. (**L**) Localization of the N protein of mutant PRRSV strains in cells. MARC-145 cells were either mock-infected or infected with reverse gene strains of TA-12 at an MOI of 0.01 for 24 h. The PRRSV N protein was detected and colored with a monoclonal antibody. Fluorescein isothiocyanate goat anti-mouse IgG is shown in green, and intracellular localization was determined based on the green signal in the merged image. (**M**) Effect of mutant PRRSV strains on IL-8 mRNA and protein level. Reverse genetic strains of TA-12 were used to infect MARC-145 cells at an MOI of 0.01, and cells and supernatants were collected at the indicated HPI. IL-8 mRNA levels were detected by qPCR. (**N**) Effect of mutant PRRSV strains on IL-8 protein level. Reverse genetic strains of TA-12 were used to infect PAMs at an MOI of 0.01, and the cell supernatants were collected at 24 HPI. IL-8 protein levels were measured by ELISA. (**O**) Role of 14-3-3γ in mutant PRRSV-induced IL-8 mRNA and protein level. 14-3-3γ was knocked down in MARC-145 cells using siRNA, followed by infection with mutant PRRSV strains. Cells were collected at 0, 6, 12, 24, and 36 HPI, and IL-8 mRNA levels were measured by qPCR. (**P**) Role of 14-3-3γ in mutant PRRSV-induced IL-8 protein level. PAMs were transfected with siRNA targeting 14-3-3γ or non-targeting control (NC) for 24 h and then infected with mutant PRRSV strains at an MOI of 0.01. The cell supernatants were collected at 24 HPI, and IL-8 protein levels were measured by ELISA. Glyceraldehyde-3-phosphate dehydrogenase (GAPDH) was used as an internal control for qPCR. Error bars represent the SD of three experimental replicates. GAPDH was used as an internal control for Western blotting. Multiplicity of infection (MOI). Enzyme-linked immunosorbent assay (ELISA). Asterisks indicate significant differences compared to controls: *P < 0.05; *P* < 0.01 (unpaired two-tailed Student’s *t*-test).

To identify the specific viral protein responsible for regulating IL-8 expression, constructs encoding all PRRSV proteins were co-transfected with an IL-8 promoter-luciferase reporter into MARC-145^si14-3-3γ^ and MARC-145^siNC^ cell lines. The luciferase reporter assay revealed that PRRSV proteins NSP4 and N upregulated IL-8 transcription by more than twofold (Fig. 6A). To further validate these findings, constructs encoding NSP4 and N were transfected into MARC-145 cells, using empty vector and NSP3 constructs as controls. The results demonstrated that only the N protein significantly upregulated IL-8 promoter activity (Fig. 6B).

To identify the specific domain of the N protein responsible for upregulating IL-8 transcription, the N protein was truncated based on its functional domains (Fig. 6C). The results indicated that the amino acid residues 11–13 (aa11–13) in the N protein are critical for upregulating IL-8 transcription (Fig. 6D through F). To determine whether this regulatory role is conserved across PRRSV strains, N proteins from different strains were co-transfected with the IL-8 promoter-luciferase reporter. The results showed that N proteins from all strains, except for the NADC30-like strain, increased IL-8 promoter activity (Fig. 6G). Sequence analysis revealed that a 12R mutation was unique to the N protein of the NADC30-like strain, while a 10N mutation was present in the N proteins of TA-02, NADC34-like, and PRRSV-1 strains (Fig. 6H). Notably, significant differences were observed between the N protein of TA-12 and those of TA-02, NADC34-like, and PRRSV-1 strains.

**Fig 7:**
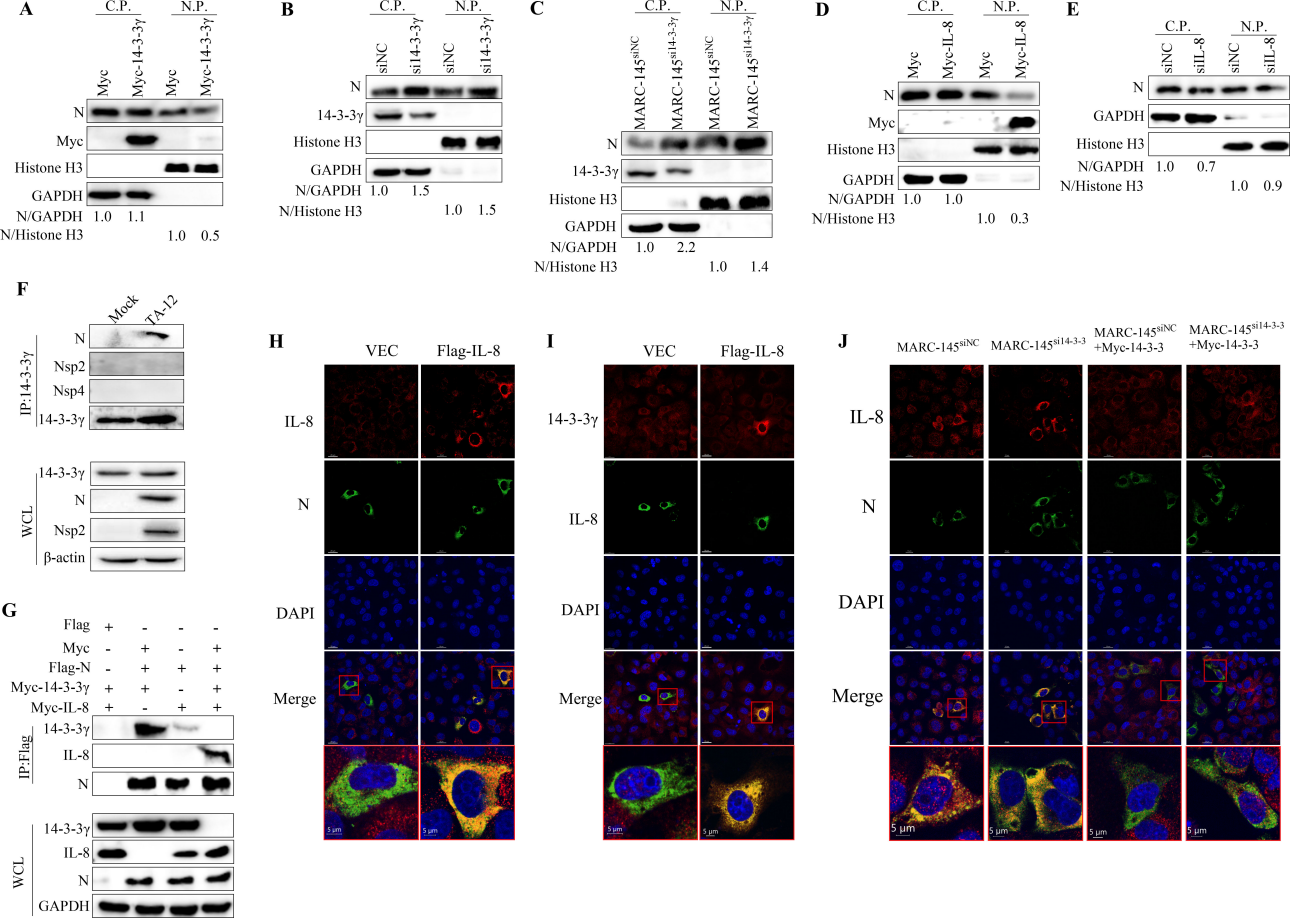
14-3-3γ and IL-8 competitively bind to N protein to modulate its nuclear distribution. (A–C) 14-3-3γ modulates the nuclear distribution of N. MARC-145 cells were transfected with Myc-14-3-3γ (**A**) or si14-3-3γ (**B**) for 24 h and then infected with TA-12. Cell samples were collected at 30 HPI, and cytoplasmic and nuclear proteins were extracted and analyzed by Western blotting. MARC-145^siNC^ and MARC-145^si14-3-3γ^ cells were infected with TA-12 for 30 h, and cytoplasmic and nuclear proteins were extracted and analyzed by Western blotting (**C**). (**D, E**) IL-8 protein modulates the nuclear distribution of N. MARC-145 cells were transfected with Myc-IL-8 (**D**) or siIL-8 (**E**) for 24 h and then infected with TA-12. Cell samples were collected at 30 HPI, and cytoplasmic and nuclear proteins were extracted and analyzed by Western blotting. (**F**) Interaction between N and 14-3-3γ. MARC-145 cells were either mock-infected or infected with HP-PRRSV TA-12 at an MOI of 0.01. Twenty-four hours later, cell lysates were pre-cleared with blank protein G resin and incubated with antibodies specific for 14-3-3γ. Proteins were analyzed by Western blotting. (**G**) Competitive interaction between N, 14-3-3γ, and IL-8. Flag-N, Myc-14-3-3γ, and Myc-IL-8 were co-transfected into human embryonic kidney 293 cell (HEK293T) cells for 24 h. Cell lysates were pre-cleared with blank protein G resin and incubated with antibodies specific for Flag. Proteins in both input samples and eluates were detected using specific antibodies. (**H**) Co-localization of IL-8 with N by immunofluorescence microscopy. MARC-145 cells were infected with TA-12 and then transfected with IL-8 plasmid. The PRRSV N protein and the IL-8 protein were detected and colored with antibodies. Co-localization was determined by the yellow signal in the merged images. (**I**) Co-localization of IL-8 with 14-3-3γ by immunofluorescence microscopy. MARC-145 cells were transfected with IL-8 plasmid. The IL-8 and 14-3-3γ proteins were detected using antibodies and subsequently stained with fluorescent secondary antibodies. Co-localization of the two proteins was determined by combining the yellow signals present in the images. (**J**) Co-localization of IL-8 with N by immunofluorescence microscopy in MARC-145^siNC^ and MARC-145^si14-3-3γ^ cells. MARC-145^siNC^ and MARC-145^si14-3-3γ^ cells were infected with TA-12. Then, they were transfected with IL-8 plasmid or IL-8 and 14-3-3γ plasmid. The PRRSV N protein and IL-8 protein were detected and stained with antibodies. Co-localization was determined by the yellow signal in the merged images. Glyceraldehyde-3-phosphate dehydrogenase (GAPDH) was used as an internal control for Western blotting of whole-cell lysates (WCLs) and cytoplasmic proteins. Histone H3 was used as an internal control for nuclear proteins. β-Actin was used as an internal control for Western blotting. C.P., cytoplasmic protein; N.P., nuclear protein. Multiplicity of infection (MOI). Asterisks indicate significant differences compared to controls: *P < 0.05; *P* < 0.01 (unpaired two-tailed Student’s *t*-test).

To investigate the functional significance of the 10N and 12R residues, the TA-12K10N, TA-12K12R, and TA-12K10N/12R mutants were generated (Fig. 6H). The results demonstrated that both TA-12K10N and TA-12K12R enhanced IL-8 promoter activity to levels comparable to TA-12 (Fig. 6I), indicating that the lysine (K) residues at positions 10 and 12 are critical for IL-8 transcriptional activation. To further confirm the role of the aa11–13 domain, four mutant strains—rTA-12/K11A, rTA-12/K12A, rTA-12/K13A, and rTA-12/K11–13A—were rescued (Fig. 6H, J, and K). Residues 11-13 (aa11-13) were identified as a functional nuclear localization signal (NLS) (16). Laser scanning confocal microscopy was used to confirm the distribution. The results demonstrated nuclear localization of the N protein exclusively in rTA-12-infected MARC-145 cells, which was absent in mutant virus-infected cells (Fig. 6L). Compared to rTA-12, all mutant strains exhibited reduced IL-8 mRNA and protein levels (Fig. 7M and N). Knockdown of 14-3-3γ did not increase IL-8 mRNA and protein levels following infection with rTA-12/K11A, rTA-12/K12A, rTA-12/K13A, or rTA-12/K11–13A (Fig. 7O and P). All these results confirm that the N protein upregulates IL-8 expression in a manner strictly dependent on its NLS domain (aa11–13) and nuclear translocation of the N protein as a prerequisite for its regulation of IL-8 transcription.

### 14-3-3γ and IL-8 competitively bind to N protein to modulate its nuclear distribution

The above results demonstrated that the N protein upregulates IL-8 transcription in a manner dependent on its nuclear distribution, while 14-3-3γ downregulates IL-8 transcription. Given the established role of 14-3-3γ proteins in modulating the cytoplasmic and nuclear distribution of target proteins, the effect of 14-3-3γ on the distribution of the N protein was investigated. Overexpression of 14-3-3γ reduced the nuclear distribution of the N protein (Fig. 7A), whereas knockdown of 14-3-3γ increased its nuclear distribution (Fig. 7B). Similarly, in MARC-145^si14-3-3γ^ cells, the nuclear distribution of the N protein was significantly higher compared to MARC-145^siNC^ cells (Fig. 7C). As shown in Fig. 5, IL-8 protein inhibits PRRSV replication. The role of IL-8 in modulating the nuclear distribution of the N protein was also examined. Overexpression of IL-8 significantly inhibited the nuclear entry of the N protein, while knockdown of IL-8 had no effect on its distribution (Fig. 7D and E). These results indicate that both 14-3-3γ and IL-8 can inhibit the nuclear distribution of the N protein. To investigate the interaction between the N protein and 14-3-3γ, TA-12 was inoculated into MARC-145 cells, and cellular proteins interacting with 14-3-3γ were immunoprecipitated using an anti-14-3-3γ antibody. Western blot analysis revealed that 14-3-3γ interacts with the N protein but not with NSP2 or NSP4 (Fig. 7F). To further explore these interactions, N, 14-3-3γ, and IL-8 were co-expressed in 293T cells. The results demonstrated that the N protein interacts with IL-8, and that 14-3-3γ and IL-8 competitively bind to the N protein (Fig. 7G). Immunofluorescence co-localization studies of N, 14-3-3γ, and IL-8 revealed distinct interaction patterns, as shown by confocal microscopy analysis, with clear co-localization observed between N and IL-8 (Fig. 7H) and between 14-3-3γ and IL-8 (Fig. 7I). Given our previous demonstration of the N-14-3-3γ interaction (33), these results collectively establish a network of pairwise interactions among all three proteins. All these results demonstrated that N protein, 14-3-3γ, and IL-8 showed pairwise interactions. Further validation in MARC-145^siNC^ versus MARC-145^si14-3-3γ^ cells demonstrated enhanced N-IL-8 co-localization following 14-3-3γ knockdown, with this increased interaction being significantly reduced in 14-3-3γ-rescued cells, supporting a competitive binding mechanism wherein 14-3-3γ modulates the N-IL-8 interaction. Collectively, these findings indicate that 14-3-3γ and IL-8 competitively bind to the N protein, modulating its nuclear distribution and thereby regulating its function.

## DISCUSSION

Cytokines play a critical role in regulating immune responses, acting as a double-edged sword in both normal physiology and disease pathology (37). Imbalanced cytokine profiles are a hallmark of viral infections, contributing to disease progression by inciting chronic inflammation and immune evasion. The manipulation or neutralization of aberrant cytokines presents a promising therapeutic approach for treating diseases, including cancer (38). Consequently, cytokine-targeted therapies—such as monoclonal antibodies, soluble receptors, or small-molecule inhibitors—offer new possibilities for patients resistant to standard pharmacological treatments (39). In this study, IL-8 was found to play distinct roles in PRRSV replication at the mRNA and protein levels. While IL-8 mRNA positively correlated with viral replication, IL-8 protein effectively inhibited PRRSV replication, highlighting its dual functionality during infection.

During PRRSV infection, IL-8 expression is significantly elevated (23). Emerging evidence suggests that IL-8 plays a complex role in viral replication. For instance, serum IL-8 levels are higher in non-persistent pigs compared to persistent pigs at 14 days post-infection (26). In other viral infections, IL-8 has been shown to suppress HIV-1 replication in macrophages through transcriptional mechanisms (40, 41), while porcine epidemic diarrhea virus induces IL-8 expression to elevate cytosolic Ca²^+^, promoting its own infection (42). Similarly, IL-8 exhibits opposing antiviral and proviral effects in hepatitis C virus infection, depending on the replication level, cellular context, and infection stage (acute vs chronic) (43). In hepatitis B virus infection, IL-8 targets the CCAAT/enhancer binding protein transcription factor, enhancing viral promoter activity and replication (44). This study revealed a striking dichotomy in the roles of IL-8 mRNA and IL-8 protein during PRRSV infection. While IL-8 mRNA levels positively correlated with PRRSV genome replication, IL-8 protein significantly inhibited viral replication. This apparent paradox suggests an evolutionary arms race between viral exploitation and host defense mechanisms. These findings redefine our understanding of cytokine function during viral infections, demonstrating that (i) cytokine mRNAs can exert functions independent of protein translation, and (ii) viruses may co-opt these non-canonical roles to facilitate replication. The precise mechanisms governing this mRNA-protein functional switch warrant further investigation.

The N protein of PRRSV localizes to the nucleus and is implicated in viral pathogenesis and host immune modulation (45). Structurally, the N protein consists of an N-terminal RNA-binding domain and a C-terminal dimerization domain. The N-terminal region (residues 1–57) is highly disordered and enriched with positive charges, facilitating RNA binding (17). This domain exhibits significant sequence variability among PRRSV strains, likely due to relaxed structural constraints (17, 46). The N protein has been shown to function as a transcriptional regulator, modulating the expression of host genes such as IL-10, IL-15, CD83 molecule, and transcription factor Dp-2 (12, 47, 48). It interacts with transcriptional regulators, including the HIC (human I-mfa domain-containing protein) homolog, suggesting its involvement in transcriptional regulation during infection (49). Additionally, the N protein interacts with other transcriptional factors, such as DHX9, DHX36, and MOV10 (50–52), and participates in viral and cellular RNA replication (53). This study provides further evidence of the N protein’s transcriptional regulatory function, particularly in upregulating IL-8 expression.

The results demonstrate that the N protein of NADC30-like strains fails to activate IL-8 transcription (Fig. 6), a finding potentially linked to their distinct clinical-epidemiological profile and pathogenic characteristics. Epidemiological data reveal that NADC30-like-infected herds typically exhibit subacute or chronic disease progression, with clinical manifestations (e.g., pyrexia, respiratory signs) generally milder than those caused by classical highly pathogenic strains, yet showing stronger association with secondary bacterial pneumonia (54) . This clinical presentation may partially reflect altered immunomodulatory capabilities: as a pivotal chemokine, IL-8 orchestrates neutrophil recruitment and mediates acute inflammatory responses. The N protein-mediated suppression of IL-8 function could lead to impaired neutrophil chemotaxis, attenuated inflammatory responses, and pathological changes characterized predominantly by lymphoid tissue damage and secondary infections rather than acute pulmonary injury.

The 14-3-3 proteins are a family of conserved regulatory molecules expressed in all eukaryotes, functioning primarily as scaffold proteins and signaling hubs (55–57). 14-3-3γ, one of the isoforms, is closely associated with inflammatory conditions (54, 58) and interacts with key signaling pathways, including NF-κB, c-Jun N-terminal kinase (JNK), protein kinase B, and mitogen-activated protein kinase (35). Previous studies have shown that 14-3-3γ interacts with PRRSV proteins (32, 33), although the specific viral targets remained unidentified.

This study elucidates the role of 14-3-3γ in modulating IL-8 expression and PRRSV replication. 14-3-3γ and IL-8 competitively interact with the N protein, regulating its nuclear distribution and thereby influencing its transcriptional activity. This competitive interaction highlights the intricate balance between host and viral factors in modulating infection outcomes.

## MATERIALS AND METHODS

### Cells and virus

MARC-145 and 293T cells were obtained from the China Center for Type Culture Collection (Wuhan, China) and maintained in Dulbecco’s modified Eagle’s medium (Gibco, Langley, OK, USA) supplemented with 10% fetal bovine serum at 37°C in a humidified 5% CO_₂_ incubator. The low-pathogenic PRRSV strain CH-1R and PK15^CD163^ cells (a PK15 cell line stably expressing CD163) were kindly provided by Dr. Qin Zhao (Northwest A&F University, China). The HP-PRRSV strain TA-12 (GenBank accession no. HQ416720), a classical low-pathogenicity strain CH-1R (PRRSV-2, lineage 5.1), NADC30-like strain TA-01 (PRRSV-2, lineage 1.8), and recombinant strain TA-02 (generated by recombination of HP-PRRSV and NADC30-like strains, with the structural proteins derived from HP-PRRSV and nonstructural proteins from NADC30-like) were previously isolated and stored in our laboratory. PAMs were isolated from five healthy 5-week-old crossbred weaned pigs (Landrace × Yorkshire) using a previously described protocol.

### Antibodies and reagents

The following antibodies were used: anti-14-3-3γ (#5522), anti-Histone H3 (#4499), and anti-Myc-Tag (#2272) from Cell Signaling Technology; anti-β-Actin (AP0060) from Bioworld Technology; anti-GAPDH (AT1749) and anti-Flag (AT0022) from Engibody Biotechnology. Monoclonal antibodies against PRRSV N protein, Nsp2, and Nsp4 were prepared in our lab.

The Porcine IL-8/CXCL8 Quantikine ELISA Kit (P8000) was purchased from R&D Systems (Switzerland). The BCA Protein Assay Kit (23225), Lipofectamine 3000 (L3000015), and Lipofectamine RNAiMAX (13778075) were obtained from Thermo Fisher Scientific. ReverTra Ace qPCR RT Kit (NLQ-101) and KOD qPCR SYBR® Mix (QKD-201T) were purchased from TOYOBO. Inhibitors PDTC (HY-18738), SB203580 (HY-10256), SP600125 (HY-12041), BAY 11-7082 (HY-13453), and puromycin (HY-K1057) were sourced from MedChemExpress (Shanghai, China). Radioimmunoprecipitation assay (RIPA) lysis buffer (P0013C), lysis buffer (P0013), dual-luciferase reporter assay kit (RG027), and cytoplasmic and nuclear protein extraction kit (P0027) were purchased from Beyotime.

### Plasmid construction and transfection

Plasmids encoding PRRSV-2 structural and nonstructural proteins were previously constructed in our laboratory using the p3×FLAG-myc-CMV-26 vector. Genes encoding 14-3-3γ (GenBank accession no. NM_012479.4) and IL-8 (GenBank accession no. NM_001032965.1) were amplified and inserted into p3×FLAG-myc-CMV-26 or pCMV-Myc vectors. Truncated and mutant forms of TA-12 N were generated by PCR and cloned into p3×FLAG-myc-CMV-26 or pEGFP-C1 vectors. All constructs were verified by Sanger sequencing and listed in Table S1. HEK293T cells were transfected using polyethylenimine (Yeasen), while MARC-145, PK-15CD163, and PAMs were transfected using Lipofectamine 3000.

### siRNA transfection

siRNAs targeting specific genes were obtained from GenePharma (Shanghai, China) and transfected into PAMs, MARC-145, or PK-15^CD163^ cells using Lipofectamine RNAiMAX according to the manufacturer’s instructions. siRNA sequences are listed in Table S2.

### Western blotting

Total protein was extracted using RIPA lysis buffer (50 mM Tris-HCl, pH 7.4, 150 mM NaCl, 1 mM EDTA, 1% NP-40, 0.5% sodium deoxycholate, 0.1% SDS, 1× protease inhibitor cocktail, and 1× phosphatase inhibitor PhosStop). Protein concentration was determined using a BCA Protein Assay Kit (Thermo Fisher Scientific). Proteins were separated by 12%–15% SDS-PAGE and transferred to polyvinylidene fluoride membranes (Millipore). After blocking with Rapid Blocking Buffer (BestEnzymes), membranes were incubated with primary antibodies overnight at 4°C, followed by secondary antibodies for 1 h at room temperature. Protein bands were visualized using an Azure 600 imaging system (Azure Biosystems).

### Co-immunoprecipitation assay

Cells were lysed in buffer supplemented with cOmplete EDTA-free Protease Inhibitor Cocktail (Roche) and rotated at 4°C for 30 min. Lysates were centrifuged at 500 × *g* for 10 min, and 500 µg of protein was immunoprecipitated with anti-Flag or anti-14-3-3γ antibodies and Protein A/G MagBeads (GenScript) overnight at 4°C. Immunoprecipitants were washed five times with lysis buffer, denatured in 5× loading buffer, and analyzed by SDS-PAGE and immunoblotting.

### Confocal microscopy

Cells were fixed with 4% paraformaldehyde (Solarbio, China) for 15 min and permeabilized with 0.1% (vol/vol) Triton X-100 (Solarbio, China) in phosphate-buffered saline. After blocking with 1% bovine serum albumin (Solarbio, China) in phosphate buffered saline for 1 h, the cells were incubated with primary antibodies for 1 h. Cells were then washed and incubated with fluorescently labeled secondary antibodies conjugated to fluorescein isothiocyanate or Cyanine 3 (Cy3). Cell nuclei were stained with 4′,6-diamidino-2-phenylindole (Invitrogen, USA). All probed cells were observed under a fluorescence microscope (Andor Dragonfly, Oxford Instruments, Oxon, UK).

### Rescue of recombinant PRRSV

The plasmid pACYC177-CMV-MCS-HDV-SV40, containing a cytomegalovirus (CMV) promoter, multiple-cloning site (MCS), hepatitis delta virus (HDV) ribozyme, and simian virus 40 (SV40) signal, was modified from the low-copy-number plasmid pACYC177. Full-length cDNA clones of HP-PRRSV TA-12 or N gene mutants (K11A, K12A, K13A, and K11–13A) (Table S1) were assembled by homologous recombination. Recombinant viruses were rescued by transfection of BHK-21 cells. Viral titers were determined by TCID_50_ assay, and growth curves were generated using GraphPad Prism 8.

### IL-8 protein analysis by ELISA

IL-8 concentrations in cell culture supernatants were measured using a Porcine IL-8 ELISA Kit (R&D Systems) according to the manufacturer’s instructions. Absorbance was measured at 450 nm.

### qRT-PCR analysis

Total RNA was extracted using TRIzol (Takara) and reverse-transcribed using the ReverTra Ace qPCR RT Kit (TOYOBO). qRT-PCR was performed using KOD qPCR SYBR Mix (TOYOBO) on a LightCycler 96 Instrument (Roche). Primers are listed in Table S1. Data were normalized to GAPDH expression and analyzed using the 2^-ΔΔCt^ method.

### Virus titer assay

Viral titers were determined by endpoint dilution assay on MARC-145 cells. Serial 10-fold dilutions of virus samples were added to 96-well plates, and titers were calculated using the Reed-Muench method.

### Development of MARC-145^siNC^ and MARC-145^si14-3-3γ^ cell lines

shRNA oligonucleotides targeting 14-3-3γ were designed using the Broad Institute shRNA Design Tool and cloned into the pLKO.1-puro lentiviral vector. Lentivirus was produced in HEK293T cells using psPAX2 and pMD2.G packaging plasmids. MARC-145 cells were transduced and selected with puromycin (10 µg/mL). Knockdown efficiency was confirmed by qRT-PCR and Western blotting.

### Cell viability assay

Cell viability was assessed using a Cell Counting Kit-8 (New Cell & Molecular Biotech) according to the manufacturer’s instructions. Absorbance was measured at 450 nm.

### Luciferase reporter assays

HEK293T, MARC-145^siNC^, and MARC-145^si14-3-3γ^ cells were transfected with pGL3-IL-8-Luc and pRL-TK plasmids using Lipofectamine 3000. Luciferase activity was measured using a dual-luciferase reporter assay kit (Beyotime).

### IL-8 protein expression and purification

pET-28a-IL-8 was transformed into *Escherichia coli* BL21 cells and induced with 1 mM isopropyl β-D-1-thiogalactopyranoside. Recombinant His-tagged IL-8 was purified using Ni-NTA resin (GenScript) and analyzed by SDS-PAGE.

### Isolation of nuclear and cytoplasmic proteins

Nuclear and cytoplasmic proteins were extracted using a cytoplasmic and nuclear protein extraction kit (Beyotime) and analyzed by Western blotting.

### Statistical analysis

Data were analyzed using one-way analysis of variance or Student’s *t*-test in SPSS 20.0 (SPSS Inc., USA). Results are expressed as mean ± SD from at least three independent experiments. A *P*-value <0.05 was considered statistically significant.

## Data Availability

All data supporting the findings of this study are included in the article and its supplemental material. Additional raw data are available from the corresponding author upon reasonable request.

## SUPPLEMENTAL MATERIAL

Supplemental tables jvi.00655-25-s0001.docx Sequences of primers and siRNA.

ASM does not own the copyrights to Supplemental Material that may be linked to, or accessed through, an article. The authors have granted ASM a non-exclusive, world-wide license to publish the Supplemental Material files. Please contact the corresponding author directly for reuse.

## References

[1] Holtkamp D, Kliebenstein J, Neumann E, Zimmerman J, Rotto H, Yoder T, Wang C, Yeske P, Mowrer C, Haley C. 2013. Assessment of the economic impact of porcine reproductive and respiratory syndrome virus on United States pork producers. J Swine Health Prod 21:72–84. doi:10.54846/jshap/754

[2] Kuhn JH, Lauck M, Bailey AL, Shchetinin AM, Vishnevskaya TV, Bào Y, Ng TFF, LeBreton M, Schneider BS, Gillis A, et al.. 2016. Reorganization and expansion of the nidoviral family Arteriviridae. Arch Virol 161:755–768. doi:10.1007/s00705-015-2672-z 26608064 PMC5573231

[3] Xu H, Li C, Li W, Zhao J, Gong B, Sun Q, Tang Y-D, Xiang L, Leng C, Peng J, Wang Q, Meng F, Yu Y, An T, Cai X, Tian Z-J, Zhang H. 2022. Novel characteristics of Chinese NADC34-like PRRSV during 2020-2021. Transbound Emerg Dis 69:e3215–e3224. doi:10.1111/tbed.14485 35182461

[4] Wang X, Zhang K, Mo Q, Chen G, Lv J, Huang J, Pang Y, Wang H, Liu W, Huang K, Min X, Ren T, Ouyang K, Chen Y, Huang W, Wei Z. 2022. The emergence and pathogenesis of recombinant viruses associated with NADC34-like strains and the predominant circulating strains of porcine reproductive and respiratory syndrome virus in southern China. Viruses 14:1695. doi:10.3390/v14081695 36016319 PMC9416154

[5] Huang B, Xu T, Luo Z, Deng L, Jian Z, Lai S, Ai Y, Zhou Y, Ge L, Xu Z, Zhu L. 2024. Prevalence and genetic diversity of PRRSV in Sichuan province of China from 2021 to 2023: evidence of an ongoing epidemic transition. Virology (Auckl) 600:110213. doi:10.1016/j.virol.2024.110213 39265448

[6] Trevisan G, Li G, Moura CAA, Coleman K, Thomas P, Zhang J, Gauger P, Zeller M, Linhares D. 2021. Complete coding genome sequence of a novel porcine reproductive and respiratory syndrome virus 2 restriction fragment length polymorphism 1-4-4 lineage 1C variant identified in Iowa, USA. Microbiol Resour Announc 10:e0044821. doi:10.1128/MRA.00448-21 34042485 PMC8213044

[7] Han J, Wang Y, Faaberg KS. 2006. Complete genome analysis of RFLP 184 isolates of porcine reproductive and respiratory syndrome virus. Virus Res 122:175–182. doi:10.1016/j.virusres.2006.06.003 16860427

[8] Dea S, Gagnon CA, Mardassi H, Pirzadeh B, Rogan D. 2000. Current knowledge on the structural proteins of porcine reproductive and respiratory syndrome (PRRS) virus: comparison of the North American and European isolates. Arch Virol 145:659–688. doi:10.1007/s007050050662 10893147 PMC7087215

[9] Fang Y, Snijder EJ. 2010. The PRRSV replicase: exploring the multifunctionality of an intriguing set of nonstructural proteins. Virus Res 154:61–76. doi:10.1016/j.virusres.2010.07.030 20696193 PMC7114499

[10] Johnson CR, Griggs TF, Gnanandarajah J, Murtaugh MP. 2011. Novel structural protein in porcine reproductive and respiratory syndrome virus encoded by an alternative ORF5 present in all arteriviruses. J Gen Virol 92:1107–1116. doi:10.1099/vir.0.030213-0 21307222 PMC3139420

[11] Snijder EJ, Meulenberg JJ. 1998. The molecular biology of arteriviruses. J Gen Virol 79 (Pt 5):961–979. doi:10.1099/0022-1317-79-5-961 9603311

[12] Liu X, Fan B, Bai J, Wang H, Li Y, Jiang P. 2015. The N-N non-covalent domain of the nucleocapsid protein of type 2 porcine reproductive and respiratory syndrome virus enhances induction of IL-10 expression. J Gen Virol 96:1276–1286. doi:10.1099/vir.0.000061 25614594

[13] Gong X, Ma T, Wang J, Cao X, Zhang Q, Wang Y, Song C, Lai M, Zhang C, Fang X, Chen X. 2023. Nucleocapsid protein residues 35, 36, and 113 are critical sites in up-regulating the Interleukin-8 production via C/EBPα pathway by highly pathogenic porcine reproductive and respiratory syndrome virus. Microb Pathog 184:106345. doi:10.1016/j.micpath.2023.106345 37714310

[14] Lee SM, Kleiboeker SB. 2005. Porcine arterivirus activates the NF-κB pathway through IκB degradation. Virology (Auckl) 342:47–59. doi:10.1016/j.virol.2005.07.034 PMC7111765 16129468

[15] Wootton SK, Rowland RRR, Yoo D. 2002. Phosphorylation of the porcine reproductive and respiratory syndrome virus nucleocapsid protein. J Virol 76:10569–10576. doi:10.1128/jvi.76.20.10569-10576.2002 12239338 PMC136587

[16] Rowland RR, Kervin R, Kuckleburg C, Sperlich A, Benfield DA. 1999. The localization of porcine reproductive and respiratory syndrome virus nucleocapsid protein to the nucleolus of infected cells and identification of a potential nucleolar localization signal sequence. Virus Res 64:1–12. doi:10.1016/s0168-1702(99)00048-9 10500278

[17] Yoo D, Wootton SK, Li G, Song C, Rowland RR. 2003. Colocalization and interaction of the porcine arterivirus nucleocapsid protein with the small nucleolar RNA-associated protein fibrillarin. J Virol 77:12173–12183. doi:10.1128/jvi.77.22.12173-12183.2003 14581554 PMC254285

[18] Lee C, Hodgins D, Calvert JG, Welch S-KW, Jolie R, Yoo D. 2006. Mutations within the nuclear localization signal of the porcine reproductive and respiratory syndrome virus nucleocapsid protein attenuate virus replication. Virology (Auckl) 346:238–250. doi:10.1016/j.virol.2005.11.005 PMC7172752 16330065

[19] Sagong M, Lee C. 2011. Porcine reproductive and respiratory syndrome virus nucleocapsid protein modulates interferon-β production by inhibiting IRF3 activation in immortalized porcine alveolar macrophages. Arch Virol 156:2187–2195. doi:10.1007/s00705-011-1116-7 21947566 PMC7086947

[20] Zhao K, Li L-W, Jiang Y-F, Gao F, Zhang Y-J, Zhao W-Y, Li G-X, Yu L-X, Zhou Y-J, Tong G-Z. 2019. Nucleocapsid protein of porcine reproductive and respiratory syndrome virus antagonizes the antiviral activity of TRIM25 by interfering with TRIM25-mediated RIG-I ubiquitination. Vet Microbiol 233:140–146. doi:10.1016/j.vetmic.2019.05.003 31176400 PMC7117424

[21] Chen X, Quan R, Guo X, Gao L, Shi J, Feng W. 2014. Up-regulation of pro-inflammatory factors by HP-PRRSV infection in microglia: implications for HP-PRRSV neuropathogenesis. Vet Microbiol 170:48–57. doi:10.1016/j.vetmic.2014.01.031 24581811

[22] Qiao S, Feng L, Bao D, Guo J, Wan B, Xiao Z, Yang S, Zhang G. 2011. Porcine reproductive and respiratory syndrome virus and bacterial endotoxin act in synergy to amplify the inflammatory response of infected macrophages. Vet Microbiol 149:213–220. doi:10.1016/j.vetmic.2010.11.006 21129861

[23] Thanawongnuwech R, Thacker B, Halbur P, Thacker EL. 2004. Increased production of proinflammatory cytokines following infection with porcine reproductive and respiratory syndrome virus and Mycoplasma hyopneumoniae. Clin Diagn Lab Immunol 11:901–908. doi:10.1128/CDLI.11.5.901-908.2004 15358650 PMC515260

[24] Van Reeth K, Labarque G, Nauwynck H, Pensaert M. 1999. Differential production of proinflammatory cytokines in the pig lung during different respiratory virus infections: correlations with pathogenicity. Res Vet Sci 67:47–52. doi:10.1053/rvsc.1998.0277 10425240 PMC7126504

[25] Liu Y, Du Y, Wang H, Du L, Feng WH. 2017. Porcine reproductive and respiratory syndrome virus (PRRSV) up-regulates IL-8 expression through TAK-1/JNK/AP-1 pathways. Virology (Auckl) 506:64–72. doi:10.1016/j.virol.2017.03.009 PMC7111726 28347884

[26] Lunney JK, Fritz ER, Reecy JM, Kuhar D, Prucnal E, Molina R, Christopher-Hennings J, Zimmerman J, Rowland RRR. 2010. Interleukin-8, interleukin-1β, and interferon-γ levels are linked to PRRS virus clearance. Viral Immunol 23:127–134. doi:10.1089/vim.2009.0087 20373993

[27] Koch AE, Polverini PJ, Kunkel SL, Harlow LA, DiPietro LA, Elner VM, Elner SG, Strieter RM. 1992. Interleukin-8 as a macrophage-derived mediator of angiogenesis. Science 258:1798–1801. doi:10.1126/science.1281554 1281554

[28] Baggiolini M, Dewald B, Moser B. 1994. Interleukin-8 and related chemotactic cytokines--CXC and CC chemokines. Adv Immunol 55:97–179. 8304236

[29] Matsushima K, Yang D, Oppenheim JJ. 2022. Interleukin-8: an evolving chemokine. Cytokine 153:155828. doi:10.1016/j.cyto.2022.155828 35247648

[30] Skov L, Beurskens FJ, Zachariae COC, Reitamo S, Teeling J, Satijn D, Knudsen KM, Boot EPJ, Hudson D, Baadsgaard O, Parren PWHI, van de Winkel JGJ. 2008. IL-8 as antibody therapeutic target in inflammatory diseases: reduction of clinical activity in palmoplantar pustulosis. J Immunol 181:669–679. doi:10.4049/jimmunol.181.1.669 18566434

[31] Fu Y, Quan R, Zhang H, Hou J, Tang J, Feng W. 2012. Porcine reproductive and respiratory syndrome virus induces interleukin-15 through the NF-κB signaling pathway. J Virol 86:7625–7636. doi:10.1128/JVI.00177-12 22573868 PMC3416278

[32] Xiao Y, Wu W, Gao J, Smith N, Burkard C, Xia D, Zhang M, Wang C, Archibald A, Digard P, Zhou E-M, Hiscox JA. 2016. Characterization of the interactome of the porcine reproductive and respiratory syndrome virus nonstructural protein 2 reveals the hyper variable region as a binding platform for association with 14-3-3 proteins. J Proteome Res 15:1388–1401. doi:10.1021/acs.jproteome.5b00396 26709850

[33] Cao S, Liu J, Ding G, Shao Q, Wang B, Li Y, Feng J, Zhao Y, Liu S, Xiao Y. 2020. The tail domain of PRRSV NSP2 plays a key role in aggrephagy by interacting with 14-3-3ε. Vet Res 51:104. doi:10.1186/s13567-020-00816-7 32811532 PMC7433210

[34] Zhou X-Y, Hu DX, Chen RQ, Chen XQ, Dong W-L, Yi C. 2017. 14-3-3 isoforms differentially regulate NFκB signaling in the brain after ischemia-reperfusion. Neurochem Res 42:2354–2362. doi:10.1007/s11064-017-2255-3 28424948

[35] Radhakrishnan VM, Martinez JD. 2010. 14-3-3gamma induces oncogenic transformation by stimulating MAP kinase and PI3K signaling. PLoS One 5:e11433. doi:10.1371/journal.pone.0011433 20628654 PMC2900177

[36] Németh ZH, Deitch EA, Szabó C, Haskó G. 2003. Pyrrolidinedithiocarbamate inhibits NF-κB activation and IL-8 production in intestinal epithelial cells. Immunol Lett 85:41–46. doi:10.1016/s0165-2478(02)00208-0 12505195

[37] Yi Ming, Li T, Niu M, Zhang H, Wu Y, Wu K, Dai Z. 2024. Targeting cytokine and chemokine signaling pathways for cancer therapy. Signal Transduct Target Ther 9:176. doi:10.1038/s41392-024-01868-3 39034318 PMC11275440

[38] Yi M, Li T, Niu M, Wu Y, Zhao Z, Wu K. 2022. TGF-β: a novel predictor and target for anti-PD-1/PD-L1 therapy. Front Immunol 13:1061394. doi:10.3389/fimmu.2022.1061394 36601124 PMC9807229

[39] Striz I, Brabcova E, Kolesar L, Sekerkova A. 2014. Cytokine networking of innate immunity cells: a potential target of therapy. Clin Sci (Lond) 126:593–612. doi:10.1042/CS20130497 24450743

[40] Xiong XX, Hu DX, Xu L, Lin H, Zhang Y, Li CY, Chen XQ. 2019. Selective 14-3-3γ upregulation promotes beclin-1-LC3-autophagic influx via β-catenin interaction in starved neurons in vitro and in vivo. Neurochem Res 44:849–858. doi:10.1007/s11064-019-02717-4 30635843

[41] Csoma E, Deli T, Kónya J, Csernoch L, Beck Z, Gergely L. 2006. Human herpesvirus 6A decreases the susceptibility of macrophages to R5 variants of human immunodeficiency virus 1: possible role of RANTES and IL-8. Virus Res 121:161–168. doi:10.1016/j.virusres.2006.05.007 16815583

[42] Guo X, Feng Y, Zhao X, Qiao S, Ma Z, Li Z, Zheng H, Xiao S. 2023. Coronavirus porcine epidemic diarrhea virus utilizes chemokine interleukin-8 to facilitate viral replication by regulating Ca^2+^ Flux. J Virol 97:e0029223. doi:10.1128/jvi.00292-23 37133374 PMC10231212

[43] Koo BCA, McPoland P, Wagoner JP, Kane OJ, Lohmann V, Polyak SJ. 2006. Relationships between hepatitis C virus replication and CXCL-8 production in vitro. J Virol 80:7885–7893. doi:10.1128/JVI.00519-06 16873245 PMC1563830

[44] Yang K, Zhu Y, Chen J, Zhou W. 2024. Interleukin-8 in HepG2 cells: Enhancing antiviral proteins in uninfected cells but promoting HBV replication in infected cells. Biochem Biophys Res Commun 734:150455. doi:10.1016/j.bbrc.2024.150455 39083972

[45] Pei Y, Hodgins DC, Lee C, Calvert JG, Welch S-KW, Jolie R, Keith M, Yoo D. 2008. Functional mapping of the porcine reproductive and respiratory syndrome virus capsid protein nuclear localization signal and its pathogenic association. Virus Res 135:107–114. doi:10.1016/j.virusres.2008.02.012 18403041

[46] Meng XJ, Paul PS, Halbur PG, Lum MA. 1995. Phylogenetic analyses of the putative M (ORF 6) and N (ORF 7) genes of porcine reproductive and respiratory syndrome virus (PRRSV): implication for the existence of two genotypes of PRRSV in the U.S.A. and Europe. Arch Virol 140:745–755. doi:10.1007/BF01309962 7794115 PMC7086766

[47] Chen X, Zhang Q, Bai J, Zhao Y, Wang X, Wang H, Jiang P. 2017. The nucleocapsid protein and nonstructural protein 10 of highly pathogenic porcine reproductive and respiratory syndrome virus enhance CD83 production via NF-κB and Sp1 signaling pathways. J Virol 91:e00986-17. doi:10.1128/JVI.00986-17 28659471 PMC5571251

[48] Zhu M, Li X, Sun R, Shi P, Cao A, Zhang L, Guo Y, Huang J. 2021. The C/EBPβ-dependent induction of TFDP2 facilitates porcine reproductive and respiratory syndrome virus proliferation. Virol Sin 36:1341–1351. doi:10.1007/s12250-021-00403-w 34138404 PMC8209777

[49] Song C, Lu R, Bienzle D, Liu HC, Yoo D. 2009. Interaction of the porcine reproductive and respiratory syndrome virus nucleocapsid protein with the inhibitor of MyoD family-a domain-containing protein. Biol Chem 390:215–223. doi:10.1515/BC.2009.028 19090724

[50] Jing H, Zhou Y, Fang L, Ding Z, Wang D, Ke W, Chen H, Xiao S. 2017. DExD/H-box helicase 36 signaling via myeloid differentiation primary response gene 88 contributes to NF-κB activation to type 2 porcine reproductive and respiratory syndrome virus infection. Front Immunol 8:1365. doi:10.3389/fimmu.2017.01365 29123520 PMC5662876

[51] Wang X, Bai J, Zhang L, Wang X, Li Y, Jiang P. 2012. Poly(A)-binding protein interacts with the nucleocapsid protein of porcine reproductive and respiratory syndrome virus and participates in viral replication. Antiviral Res 96:315–323. doi:10.1016/j.antiviral.2012.09.004 22985629

[52] Zhao K, Li L-W, Zhang Y-J, Jiang Y-F, Gao F, Li G-X, Yu L-X, Zhao W-Y, Shan T-L, Zhou Y-J, Tong G-Z. 2018. MOV10 inhibits replication of porcine reproductive and respiratory syndrome virus by retaining viral nucleocapsid protein in the cytoplasm of Marc-145 cells. Biochem Biophys Res Commun 504:157–163. doi:10.1016/j.bbrc.2018.08.148 30172377

[53] Liu L, Tian J, Nan H, Tian M, Li Y, Xu X, Huang B, Zhou E, Hiscox JA, Chen H. 2016. Porcine reproductive and respiratory syndrome virus nucleocapsid protein interacts with Nsp9 and cellular DHX9 to regulate viral RNA synthesis. J Virol 90:5384–5398. doi:10.1128/JVI.03216-15 27009951 PMC4934760

[54] Li C, Zhuang J, Wang J, Han L, Sun Z, Xiao Y, Ji G, Li Y, Li X, Tian K. 2016. Outbreak investig Investigation of NADC30-Like PRRSV in south-ein South-East China. Transbound Emerg Dis 63:474–479. doi: 10.1111/tbed.12530 27292168 10.1111/tbed.12530

[55] Dougherty MK, Morrison DK. 2004. Unlocking the code of 14-3-3. J Cell Sci 117:1875–1884. doi:10.1242/jcs.01171 15090593

[56] Fu H, Subramanian RR, Masters SC. 2000. 14-3-3 proteins: structure, function, and regulation. Annu Rev Pharmacol Toxicol 40:617–647. doi:10.1146/annurev.pharmtox.40.1.617 10836149

[57] Liu J, Cao S, Ding G, Wang B, Li Y, Zhao Y, Shao Q, Feng J, Liu S, Qin L, Xiao Y. 2021. The role of 14‐3‐3 proteins in cell signalling pathways and virus infection. J Cellular Molecular Medi 25:4173–4182. doi:10.1111/jcmm.16490 PMC8093981 33793048

[58] Kilani RT, Maksymowych WP, Aitken A, Boire G, St-Pierre Y, Li Y, Ghahary A. 2007. Detection of high levels of 2 specific isoforms of 14-3-3 proteins in synovial fluid from patients with joint inflammation. J Rheumatol 34:1650–1657. 17611984

