# Supplementary material for: The mRNA and protein of IL-8 oppositely regulate PRRSV replication via nucleoprotein and 14-3-3γ
Source: J Virol. 2025 Jul 24;99(8):e00655-25. doi: 10.1128/jvi.00655-25 (PMC12363195; doi:10.1128/jvi.00655-25)
Supplement: Supplemental tables — Sequences of primers and siRNA. [file jvi.00655-25-s0001.docx]

| **Table S1 A list of primers for constructs and qPCR** | | | | |
| --- | --- | --- | --- | --- |
| Vectors | | Gene | Forward (5’-3’) | Reverse (5’-3’) |
| pCMV-HA | | NSP1α | GATCAGATCTCTATGTCTGGGATACTTGATCG | CCCGCGGCCGCTTACATAGCACATCAAAAGGGC |
|  |  | NSP1β | GATCAGATCTCTATGGCTGACGTCTATGACATTGG | CCCGCGGCCGCTTACCCGAAGACCATTAACTTGC |
|  |  | NSP2 | GATCAGATCTCTATGGCCGGAAAGAGAGCAAGG | CCCGCGGCCGCTTATCCCCCTGAAGGCTTGG |
|  |  | NSP3 | GATCAGATCTCTATGGGCCCACAACCTCATTGCTGCC | CCCGCGGCCGCTTACTCAAGGAGGGACCCGAGCTG |
|  |  | NSP4 | GATCAGATCTCTGGTGCTTTCAGAACTCAA | CCCGCGGCCGCTTATTCCAGTTCGGGTTTGGC |
|  |  | NSP5 | CCGCTCGAGGTATGGGAGGCCTTTCTACAGTTCAAC | CCCGCGGCCGCTTACTCGCAAAGTATCGAAGAAG |
|  |  | NSP6 | GATCAGATCTCTATGGGAAAGTTGAGGGAAGGGGTG | CCCGCGGCCGCTTACTCATGATTTATCCCGCAGG |
|  |  | NSP7 | GATCAGATCTCTATGTCGCTGACTGGTGCCCTC | CCCGCGGCCGCTTATTCCCACTGAGCTCTTCTATTC |
|  |  | NSP8 | GATCAGATCTCTATGGCCGCCAAGCTTTCCGTG | CGGCGGCCGCCTAGCAGTTTAAACTGCTCC |
|  |  | NSP9 | GATCAGATCTCTATGGCCGCCAAGCTTTCCGTG | GGGCGGCCGCTTACTCATGATTGGACCTGAGTT |
|  |  | NSP10 | CCGCTCGAGGTATGGGGAAGAAGTCCAGAATGTGC | CCCGCGGCCGCTTATTCCAGATCTGCGCAAATAGC |
|  |  | NSP11 | GATCAGATCTCTATGGGGTCGAGCTCCCCG | CCCGCGGCCGCTTATTCAAGTTGAAAATAGGCCGTC |
|  |  | NSP12 | GATCAGATCTCTATGGGCCGCCATTTTACCTGGT | CCCGCGGCCGCTCAATTCAGGCCTAAAGTTGG |
|  |  | GP2 | CAGATTACGCTCTTATGGCCATGAAATGGGGTCTATGC | GACCGAATTCGGGCCTCCATTCACCATGAGTTCAAAAG |
|  |  | GP3 | CAGATTACGCTCTTATGGCCATGGCTAATAGCTGTCCAT | GACCGAATTCGGGCCTCCATCTATCGCCGTGCGGCACT |
|  |  | GP4 | CAGATTACGCTCTTATGGCCATGGCTGCGCCCTTTCTT | GACCGAATTCGGGCCTCCATTCAAATTGCCAGTAGGATGGC |
|  |  | GP5 | CAGATTACGCTCTTATGGCCATGTTGGGGAAGTGCTTGAC | GACCGAATTCGGGCCTCCATCTAGAGACGACCCCATTGTTCC |
|  | | M | CAGATTACGCTCTTATGGCCATGGGGTCGTCTCTAGACG | GACCGAATTCGGGCCTCCATTTATTTGGCATATTTAACAAGG |
|  | | N | CAGATTACGCTCTTATGGCCATGCCAAATAACAACGGC | GACCGAATTCGGGCCTCCATTCATGCTGAGGGTGATGCTGT |
| p3×FLAG | | 14-3-3γ | ACAAGGATGACGATGACAAGATGGTGGACCGCGAGCAA | GATGAATTCGCGGCCGCAAGTTAATTGTTGCCTTCGCCGC |
| -myc- | | IL-8 | ACAAGGATGACGATGACAAGATGACTTCCAAACTGGC | GATGAATTCGCGGCCGCAAGTTACTGCTGTTGTTGTTGCTTC |
| CMV-26 | | IL-8(TGA) | CAAGTGAACTTCCAAGCTGGCGGTGG | GCTTGGAAGTTCACTTGTCATCGTCATCCTTGTAATCG |
|  | | IL-8(GCA) | TGACAAGGCAACTTCCAAGCTGGCGGTGG | TGGAAGTTGCCTTGTCATCGTCATCCTTGTAATCG |
|  | | NSP2 | ACAAGGATGACGATGACAAGATGGCCGGAAAGAGAGC | ATGAATTCGCGGCCGCAAGTTATCCCCCTGAAGGCTTGGA |
|  | | NSP3 | ACAAGGATGACGATGACAAGATGGGCCCACACCTCATT | ATGAATTCGCGGCCGCAAGTTACTCAAGGAGGGACCCGAGC |
|  | | NSP4 | ACAAGGATGACGATGACAAGATGGGTGCTTTCAGAAC | ATGAATTCGCGGCCGCAAGTTATTCCAGTTCGGGTTTGGCA |
|  | | N1-95 | ACAAGGATGACGATGACAAGATGCCAAATAACAACGGC | GATGAATTCGCGGCCGCAAGTTATGAATCTGACAGGGCACAA |
|  | | N1-68 | ACAAGGATGACGATGACAAGATGCCAAATAACAACGGC | GATGAATTCGCGGCCGCAAGTTAGGTAAAGTGATGCCTGACG |
|  | | TA-12/N | ACAAGGATGACGATGACAAGATGCCAAATAACAACGGC | GATGAATTCGCGGCCGCAAGTCATGCTGAGGGTGATGCTGT |
|  | | TA-01/N | ACAAGGATGACGATGACAAGATGCCAAATAACAACGGC | GATGAATTCGCGGCCGCAAGTCATGCTGAGGGTGACGTTGT |
|  | | TA-02/N | ACAAGGATGACGATGACAAGATGCCAAATAACAACGGC | GATGAATTCGCGGCCGCAAGTCATGCTGAGGGTGACGTCG |
|  | | CH-1R/N | ACAAGGATGACGATGACAAGATGCCAAATAACAACGGC | GATGAATTCGCGGCCGCAAGTCATGCTGAGGGTGATGCTGT |
|  | | NADC34/N | ACAAGGATGACGATGACAAGATGCCAAATAACAGCGGC | GATGAATTCGCGGCCGCAAGTCATGCTGAGGGTGACGTCG |
|  | | PRRSV-1/N | ACAAGGATGACGATGACAAGATGGCCGGTAAAAACCAG | GATGAATTCGCGGCCGCAAGTTAATTTACACCCTGATTGG |
|  | | TA-12K10N | CGGCAAGCAGCAAAATAAAAAGAAGGGGAATGGCCA | TATTTTGCTGCTTGCCGTTGT |
|  | | TA-12K12R | GAAAAGGAAGGGGAATGGCCAGCC | CATTCCCCTTCCTTTTCTTTTGCTGCTTGCCG |
|  | | TA12  K10N/12R | TAAAAGGAAGGGGAATGGCCAGCC | CCATTCCCCTTCCTTTTATTTTGCTGCTTGCCGTTGT |
|  | | N/K11A | GCAAAAGGCAAAGAAGGGGAATGGCCAGC | CCTTCTTTGCCTTTTGCTGCTTGCCGTTG |
|  | | N/K12A | GAAAGCAAAGGGGAATGGCCAGCCA | CATTCCCCTTTGCTTTCTTTTGCTGCTTGCCG |
|  | | N/K13A | GAAAAAGGCAGGGAATGGCCAGCCAGTC | CATTCCCTGCCTTTTTCTTTTGCTGCTTGCCG |
|  | | N/K11-13A | AGCAAAAGGCAGCAGCAGGGAATGGCCAGCCAGTC | TGCTGCTGCCTTTTGCTGCTTGCCGTTGTTAT |
| pEGFP-C1 | | N | CTCAAGCTTCGAATTCTGCAATGCCAAATAACAACGGC | GGGCCCGCGGTACCGTCGACTCATGCTGAGGGTGATGCTGT |
|  | | N1-51 | CTCAAGCTTCGAATTCTGCAATGCCAAATAACAACGGC | GGGCCCGCGGTACCGTCGACTTACTTCTCCGGGTTTTTCTTCC |
|  | | N1-41 | CTCAAGCTTCGAATTCTGCAATGCCAAATAACAACGGC | GGGCCCGCGGTACCGTCGACTTACGGTCCCTTGCCTCTGG |
|  | | N1-31 | CTCAAGCTTCGAATTCTGCAATGCCAAATAACAACGGC | GGGCCCGCGGTACCGTCGACTTAGGCGATGATCTTACCCAGC |
|  | | N1-26 | CTCAAGCTTCGAATTCTGCAATGCCAAATAACAACGGC | GGGCCCGCGGTACCGTCGACTTACAGCATTTGGCACAGCTG |
|  | | N1-21 | CTCAAGCTTCGAATTCTGCAATGCCAAATAACAACGGC | GGGCCCGCGGTACCGTCGACTTACTGATTGACTGGCTGGCC |
|  | | N1-16 | CTCAAGCTTCGAATTCTGCAATGCCAAATAACAACGGC | GGGCCCGCGGTACCGTCGACTTAGCCATTCCCCTTCTTTTTCT |
|  | | N1-15 | CTCAAGCTTCGAATTCTGCAATGCCAAATAACAACGGC | GGGCCCGCGGTACCGTCGACTTAATTCCCCTTCTTTTTCTTTT |
|  | | N1-14 | CTCAAGCTTCGAATTCTGCAATGCCAAATAACAACGGC | GGGCCCGCGGTACCGTCGACTTACCCCTTCTTTTTCTTTTGC |
|  | | N1-13 | CTCAAGCTTCGAATTCTGCAATGCCAAATAACAACGGC | GGGCCCGCGGTACCGTCGACTTACTTCTTTTTCTTTTGCTGC |
|  | | N1-12 | CTCAAGCTTCGAATTCTGCAATGCCAAATAACAACGGC | GGGCCCGCGGTACCGTCGACTTACTTTTTCTTTTGCTGCTTGC |
|  | | N1-11 | CTCAAGCTTCGAATTCTGCAATGCCAAATAACAACGGC | GGGCCCGCGGTACCGTCGACTTATTTCTTTTGCTGCTTGCCG |
| pCMV- | | IL-8 | ACCTGCTTATGGCCATGGAGATGACTTCCAAACTGGCTG | TCGGTCGACCGAATTCGGGCTTACTGCTGTTGTTGTTGCTTC |
| Myc | | 14-3-3γ | ACCTGCTTATGGCCATGGAGATGGTGGACCGCGAGCAA | TCGGTCGACCGAATTCGGGCTTAATTGTTGCCTTCGCCGC |
| pET-28a | | IL-8(monkey) | CATCACAGCAGCGGCCTGGAAGGTGCAGTTTTGCCAAG | GGTGGTGCTCGAGTGCGGCCTTATGAATTTTGACTCTCAGC |
|  | | IL-8(swine) | CATCACAGCAGCGGCCTGGCGGTTCTGGCAAGAGTAAG | GGTGGTGCTCGAGTGCGGCCTTACTGCTGTTGTTGTTGCTTC |
| **The primers sequences for qPCR** | | | | |
| Monkey genes | Gene | | Forward (5’-3’) | Reverse (5’-3’) |
|  | GAPDH | | ACCCACTCTTCCACCTTCGACGCT | TGTTGTTGTAGCCAAATTCG |
|  | 14-3-3γ | | CGAGAAGAAGATAGAGATGGT | TCTCGTGGGCTTCGCTGT |
|  | IL-1β | | CCCTAAACAGATGAAGTGCTCC | AAGGTGCTCAGGTCATTGTC |
|  | IL-10 | | CCACGACCCAGACATCAAG | ﻿TCACTCATGGCTTTGTAGACG |
|  | IL-8 | | CGTACTCCAAACCTTTCCA | CCACTCTCAATCACTCTCAG |
|  | TNF-α | | GCCATCATCATCATCATCAC | AGCAGGAAGGAGAAGAGG |
|  | IFN-α | | GGCTTGACACTCCTGGTACAAATGAG | CAGCACATTGGCAGAGGAAGACAG |
|  | IFN-γ | | GAGACCATCAAGGAAGACATT | CGACAGTTCAGCCATCAC |
|  | c-Fos | | GAGATGTCTGTGGCTTCC | ATGCTGCTGATGCTCTTG |
|  | c-Jun | | CAAGAACTCGGACCTCCT | CCTCCTGCTCATCTGTCA |
| Swine genes | GAPDH | | CCTTCCGTGTCCCTACTGCCAAC﻿ | GACGCCTGCTTCACCACCTTCT |
|  | 14-3-3γ | | GCCATCGCCGAGCTTGACAC | CACCGTCGTCGTCTTGCTGATC |
|  | IL-8 | | TCCAAACTGGCTGTTGCCTT | ACAGTGGGGTCCACTCTCAA ﻿ |
|  | c-Fos | | CGAAGGGAAAGGAATAAGATG | TCAAGGGAAGCCACAGACA |
|  | c-Jun | | AGGCGGAGAGGAAGCGTATGAG | CTGAGCATGTTGGCGGGTGGAC |
| *PRRSV* | N | | AGATCATCGCCCAACTAAAC | GACACAATTGCCGCTCACTA |

| **Table S2 A list of siRNAs** | | | |
| --- | --- | --- | --- |
| Genes names | Nucleate site | Sequence 5′-3′ | Antisense 5'-3' |
| NC |  | UUCUCCGAACGUGUCACGUTT | ACGUGACACGUUCGGAGAATT |
| Monkey genes |  |  |  |
| 14-3-3γ | 124 | GCCACUGUCGAAUGAGGAATT | UUCCUCAUUCGACAGUGGCTT |
|  | 375 | GCAAAGUGUUCUACCUGAATT | UUCAGGUAGAACACUUUGCTT |
| IL-8 | 101 | GAGUGCAUAAAGACGUACUTT | AGUACGUCUUUAUGCACUCTT |
|  | 258 | GGGUUGUGGAGAAGUUUGUTT | ACAAACUUCUCCACAACCCTT |
| c-Fos | 376 | GGGUAAGGUGGAACAGUUATT | UAACUGUUCCACCUUACCCTT |
|  | 858 | GCUCUGUACCAGACAUGGATT | UCCAUGUCUGGUACAGAGCTT |
| c-Jun | 220 | CGAGCUGGAGCGCCUGAUATT | UAUCAGGCGCUCCAGCUCGTT |
|  | 922 | GGCACAGCUUAAACAGAAATT | UUUCUGUUUAAGCUGUGCCTT |
| Swine genes | | | |
| IL-8 | 135 | CCAAAUUUAUCAAGGAACUTT | AGUUCCUUGAUAAAUUUGGTT |
|  | 253 | GCAGAAGGUUGUACAGAUATT | UAUCUGUACAACCUUCUGCTT |
